# Stimulation of Angiotensin II Receptor Subtype 2 Reduces Preeclampsia-like Symptoms in a Mouse Model of Preeclampsia

**DOI:** 10.3390/cimb46090579

**Published:** 2024-09-02

**Authors:** Keiichi Matsubara, Yuko Matsubara, Yuka Uchikura, Takashi Sugiyama

**Affiliations:** 1 Department of Regional Pediatrics and Perinatology, Ehime University Graduate School of Medicine, Shitsukawa, Toon 791-0295, Ehime, Japan; 2 Department of Obstetrics and Gynecology, Ehime University Graduate School of Medicine, Shitsukawa, Toon 791-0295, Ehime, Japan; takeyu@m.ehime-u.ac.jp (Y.M.); yuka.itani@gmail.com (Y.U.); sugiyama.takashi.fj@ehime-u.ac.jp (T.S.)

**Keywords:** angiotensin II receptor subtype 2, CD40 ligand, compound **21**, hypertension, preeclampsia, proteinuria

## Abstract

Angiotensin II (AngII) receptor subtype 1 (AT1R) is involved in the pathogenesis of preeclampsia (PE). Angiotensin II receptor subtype 2 (AT2R) can antagonize the effects of AT1R, but its effects during pregnancy are not known. We investigated the effect of AT2R on the pathogenesis of PE using a mouse model and recently developed AT2R agonist (compound **21** [C**21**]). Blastocysts collected from pregnant imprinting control region (ICR) mice were incubated with adenovirus containing the CD40L gene and transferred into the uterine horns of pseudo-pregnant ICR mice to express PE-like features. Osmotic pumps were placed subcutaneously on the dorsal side with C**21** or saline. C**21** reduced the plasma soluble fms-like tyrosine kinase 1 (sFlt-1) concentration, ameliorating hypertension. The splenic T and B cell profiles in model mice were analyzed by flow cytometry. The gated percentage of IFN-γ-positive Th cells was significantly increased and the percentage of plasma cells in B cells was significantly decreased; however, the percentages were not altered by C**21**. sFlt-1 and soluble endoglin concentrations in plasma were measured with an enzyme-linked immunosorbent assay, and sFlt-1 was reduced. C**21** could become a candidate PE drug as it ameliorated the pathophysiology of PE as a result of decreased production of sFlt-1.

## 1. Introduction

Preeclampsia (PE) is characterized by hypertension, renal damage, thrombocytopenia, and liver dysfunction, and is associated with maternal and fetal death [1,2]. Its pathophysiology is thought to be initiated by failures in the uterine immunological acceptance of trophoblast invasion [3]. The focal immune system works to maintain pregnancy through immunosuppression. In normal pregnancy, decidual antigen-presenting cells bind to histocompatibility locus antigen G expressed on trophoblasts, leading to immune tolerance [4]. Half of the genes in the fertilized egg are of paternal origin and foreign to the mother. Therefore, if the immune tolerance does not work in early pregnancy, the invasion and proliferation of extravillous trophoblasts derived from fertilized eggs toward spiral arteries is impaired, resulting in reduced remodeling of spiral arteries. As a result, the maternal blood supply to the intervillous space is reduced, and placentation by angiogenesis and trophoblast proliferation is inhibited [5,6]. Poor placentation and uteroplacental hypoxia, in turn, can lead to the production of large amounts of anti-angiogenic factors (e.g., soluble fms-like tyrosine kinase 1 [sFlt-1] and soluble endoglin [sEng]) and their secretion into the general circulation in mid-gestation, resulting in the occurrence of PE. The impairment of maternal angiogenesis and vascular endothelial function can result in multiple organ damage, hypertension, and proteinuria via the promotion of vasoconstriction and coagulopathy [7]. This pathophysiological course suggests that abundant neovascularization at the implantation site is essential for placental development and the reduction of uterine vascular resistance early in pregnancy.

Cluster of differentiation 40 (CD40), a member of the tumor necrosis factor (TNF) receptor superfamily, and CD40 ligand (CD40L), a glycoprotein related to TNF-α, are expressed on the surfaces of activated T cells, macrophages, and endothelial cells [8]. CD40/CD40L signaling is essential for the initiation of immune and inflammatory processes [9]. We developed a mouse model of PE in which CD40L-induced immune activation affects embryo implantation and demonstrated that CD40L-induced T-helper (Th) cell type-1 differentiation during embryo implantation may play a critical role in the pathogenesis of PE-like presentation [10].

The renin–angiotensin system (RAS) plays an important role in the maintenance of normotension. Angiotensin II (AngII) is an active hormone of the RAS that plays key roles in the regulation of blood pressure (BP), vasoconstriction, coagulation, sodium homeostasis, and inflammatory responses through the activation of angiotensin II receptor subtypes 1 and 2 (AT1R and AT2R, respectively) [11]. In normal pregnancy, vascular reactivity to AngII decreases early, and refractoriness to AngII is involved in the decrease in BP mid-pregnancy [12,13,14]. Pregnant women who subsequently develop PE are exquisitely sensitive to the pressor effects of AngII, mediated by AT1R and resulting in vasoconstriction [12,15,16]. Thus, AT1R is thought to play an important role in the pathogenesis of PE with the maintenance of normal pregnancy [17]. Activated AT1R receptors induce pro-inflammation with increased reactive oxygen species production [18,19]. Angiotensin receptor blockers (ARBs) and angiotensin-converting enzyme inhibitors (ACEIs) have been shown to reduce BP and exert renoprotective effects by inhibiting AT1R, suggesting an advantage in the treatment of PE, but they are contraindicated in pregnant women due to ACEI/ARB fetopathy development [20,21]. The fetopathy results in oligohydramnios during pregnancy, severe renal failure, hypotension, and respiratory compromise immediately after birth [22].

AT2R counteracts most of the effects of AT1R by enhancing nitric oxide production via the promotion of vascular endothelial nitric oxide synthase activity through bradykinin B2 receptor (B2R) activation [23], resulting in the inhibition of cell proliferation and differentiation and reduction of vasoconstriction, inflammation, and oxidative stress [24,25]. AT1R and AT2R are highly expressed during pregnancy [26], whereas AT1R/AT2R expression is increased in PE (as with postmenopausal aging) [27,28], possibly leading to hypertension and renal dysfunction. Maternal BP has been reported to decrease mid-pregnancy in AT1a-knockout mice and increase in late pregnancy in AT2R-knockout mice [29,30]. During pregnancy, maternal serum estrogen can reduce the vascular AT1R/AT2R ratio and vascular refractoriness to AngII to maintain uteroplacental circulation [31,32]. The balance between AT1R and AT2R may be key to the regulation of vascular tone during pregnancy.

A recently developed AT2R agonist (compound **21** [C**21**]) [33] has been shown to activate AT2R, resulting in protective effects for the lungs, brain, and other organs [34,35,36,37]. Currently, the most ongoing trial on C**21** is the ASPIRE phase 2b trial being conducted for Idiopathic Pulmonary Fibrosis (IPF). This trial will evaluate whether forced vital capacity (FVC) will be improved after 52 weeks of treatment with C**21**. In IPF, type 2 alveolar epithelial cells become dysfunctional and lose their ability to repair and maintain alveolar integrity. As a result, they stimulate fibroblasts, causing pulmonary fibrosis. AT2R is highly expressed in type 2 alveolar epithelial cells. In IPF, ATRAG binds to and activates the AT2R on type 2 alveolar epithelial cells and activates it. AT2R activation promotes alveolar repair and maintenance of alveolar integrity. Finally, the fibrosis is reduced and the lung capacity is repaired [34,38]. AT2R stimulation via C**21** administration may improve the pathogenesis of PE by suppressing responses to increased reactive oxygen species and inflammatory cytokine expression [25]. In this study, we administered C**21** to PE model mice to evaluate whether it improves the phenotype of PE. We hypothesized that C**21** would have a good effect on maternal hypertension.

## 2. Materials and Methods

### 2.1. Animals

The Animal Care and Use Committee of Ehime University approved all procedures performed in this study (05HE36-16). The mouse model of PE was established using a previously described method [10]. Briefly, blastocysts were obtained from the uterine horns of mice with hyperstimulated ovaries (imprinting control region mice, 8–12 weeks old; CLEA Japan, Tokyo, Japan), incubated in complete human tubal fluid medium (Irvine Scientific, Santa Ana, CA, USA), and infected with adenoviral vectors (500 PFU) containing the CD40L gene or the β-galactosidase (LacZ) control gene. A nonpathogenic adenoviral vector plasmid (ad5CMVK-p16) was prepared by inserting the hCD40L gene (kindly provided by Dr. Fukushima, Eisai Co., Ltd., Tsukuba, Japan) or LacZ cDNA (kindly provided by Dr. Hamada, Ehime University, Matsuyama, Ehime, Japan). A few female mice were mated with vasectomized males, and the morning after the day of vaginal plug detection in these pseudo-pregnant mice was designated embryonic day (E) 0.5. Vector-infected embryos were transferred into the uterine horns (five embryos/horn) of the pseudo-pregnant mice. Pregnancy outcomes were assessed by measuring the weights of live pups delivered on E17.5.

### 2.2. C**21** Delivery

Pregnant mice receiving CD40L or null adenovirus at E0.5 were surgically implanted with subcutaneous osmotic minipumps (model 1002; Alzet. Osmotic Pumps, Cupertino, CA, USA) containing C**21** (0.43 mg/kg/d; C**21** was kindly provided by Vicore Pharma, Gothenburg, Sweden) diluted in sterile saline. The pumps were filled and implanted according to the manufacturer’s instructions.

### 2.3. Physiological Measurements

The BP was measured using the tail-cuff method with a BP-98E device (Softron Co., Ltd., Tokyo, Japan) every morning from E8.5 to the day of delivery (E17.5). The systolic blood pressure (SBP) was measured at least three times and the mean was recorded. After E8.5, the mice were placed in individual metabolic cages, and urine samples were collected automatically for 24 h. Urinary total albumin concentrations were measured using an enzyme-linked immunosorbent assay (ELISA) kit (Albuwell M; Exocell Inc., Philadelphia, PA, USA), and total creatinine concentrations were measured using a chemical assay kit (Creatinine Companion; Exocell Inc.) according to the manufacturer’s instructions. Urinary albumin concentrations were expressed as albumin/creatinine ratios (mg/g).

### 2.4. Flow Cytometric Analyses

Euthanasia was performed by cervical dislocation, and the spleens were retrieved. The spleens were dissected from the mice with forceps, and spleen cells were dispersed in Hank’s balanced salt solution (Life Technologies, Carlsbad, CA, USA). For the detection of intracellular cytokines, the cells were incubated in Roswell Park Memorial Institute-1640 medium (Life Technologies) containing brefeldin A with or without monensin (Sigma-Aldrich, Inc., St. Louis, MO, USA) or with a mixture of monensin, phorbol 12-myristate 13-acetate (Sigma), and ionomycin (Sigma) (4 h, 37 °C). Cells were then incubated for 15 min at room temperature (RT) with antibodies specific for the cell surface molecules CD4 (conjugated with peridinin chlorophyll, 1:200; Bioss Inc., Woburn, MA, USA) or isotypic control immunoglobulin G (IgG). For intracellular immunolabeling, cells were fixed and permeabilized with Intraprep^®^ reagent buffer (BD Biosciences, San Jose, CA, USA) for 20 min at RT. In addition, cells were labeled with fluorescein isothiocyanate (FITC)-conjugated anti-mouse interferon-γ (IFN-γ) antibody (1:200; Immunotec Inc., Vaudreuil-Dorion, QC, Canada) and phycoerythrin-conjugated anti-mouse interleukin-4 (IL-4) antibody (1:200; Immunotec Inc.) for 15 min at RT. Data were collected using a FACScan cytometer (BD Biosciences) and analyzed using CellQuest software ver. 8.0 (BD Biosciences). Results are expressed as percentages of total immunopositive leukocytes (% gated) and the mean fluorescence intensity of positive cells (Supplementary Figure S1).

### 2.5. B Cell Subset Analysis

Spleen cells were cultured with Fc receptor-blocking reagent (Miltenyi Biotec, Bergisch Gladbach, Germany) at 4 °C for 10 min and resuspended in phosphate-buffered saline with 1% fetal calf serum. Appropriate combinations of fluorochrome-conjugated antibodies were used to evaluate B cell subsets: control (CD19-positive), naive (CD19-positive, CD27-negative, IgD-positive), memory (CD19-positive, CD27-positive, CD24-positive, CD10-negative), transitional (CD19-positive, CD27-negative, CD24-positive, CD10-positive, CD38-positive), and plasma (CD38-positive, CD138-positive, CD27-positive, CD19-positive, IgD-negative) cells (Supplementary Figure S2).

Cells were collected using a BD Fortessa X-20 cell analyzer (Beckman Coulter, Inc., Brea, CA, USA) and analyzed using FlowJo ver. 8 (FlowJo, LLC, Ashland, OR, USA). CD19 was conjugated with phycoerythrin-Cy5.5 (1:200; RM7718, Invitrogen, Waltham, MA, USA), CD27 was conjugated with FITC (1:200; A16183, Invitrogen), CD24 was conjugated with phycoerythrin (1:200; A15836, Invitrogen), CD10 was conjugated with Alexa 647 (1:200; BS-0527R-A647, Bioss Inc.), CD38 was conjugated with Pacific Blue (1:200; A15393, Invitrogen), CD138 was conjugated with phycoerythrin-Cy7 (1:200; 142513, Biolegend, San Diego, CA, USA), and IgD was conjugated with Alexa 700 (1:200; 405730, Biolegend).

### 2.6. ELISA

Blood samples were obtained by cardiac puncture and centrifuged immediately to obtain plasma samples. Plasma levels of sFlt-1 and sEng were measured using Quantikine ELISA kits (R&D Systems, Inc., Minneapolis, MN, USA) and an iMark multiplate reader (Bio-Rad, Hercules, CA, USA). Standard, control, and plasma samples (100 µL/well) were diluted with assay diluent in microplate wells. The plates were then incubated for 2 h at RT, washed, and incubated with vascular endothelial growth factor receptor 1 (VEGFR1) or endoglin conjugate for another 2 h at RT. After washing, the plates were developed with substrate solution for 30 min at RT in the dark. The reaction was stopped, and absorbance was measured at 450 nm (primary wavelength) and 540 nm (secondary wavelength). The absorbance at 540 nm was subtracted from that at 450 nm. sFlt-1 and sEng concentrations were calculated from standard curves generated in Microplate Manager^®^ 6 (version 6.3: Bio-Rad). All values were confirmed in duplicate.

### 2.7. Statistical Analysis

For BP variability, repeated measure two-way analysis of variance (ANOVA) was performed to confirm a significant difference with respect to the effect of C**21** on sBP (*p* = 0.009), then the means of the CD40L (*n* = 9) and C**21** (*n* = 9) groups at each time point were analyzed using one-way ANOVA. There were no missing values in the measurements, and the number of samples in each group analyzed was consistent and balanced. For urine albuminuria, statistical significance was determined using one-sample Kolmogorov–Smirnov test. Other data were subjected to one-way ANOVA for statistical analysis. As this was an exploratory study, multiplicity of data was not considered. All statistical procedures were performed using SPSS ver. 26 (IBM, SPSS, Inc., Chicago, IL, USA). Data are expressed as means ± standard errors. Significant differences were considered at *p* < 0.05.

## 3. Results

### 3.1. BP and Proteinuria

**Figure 1:**
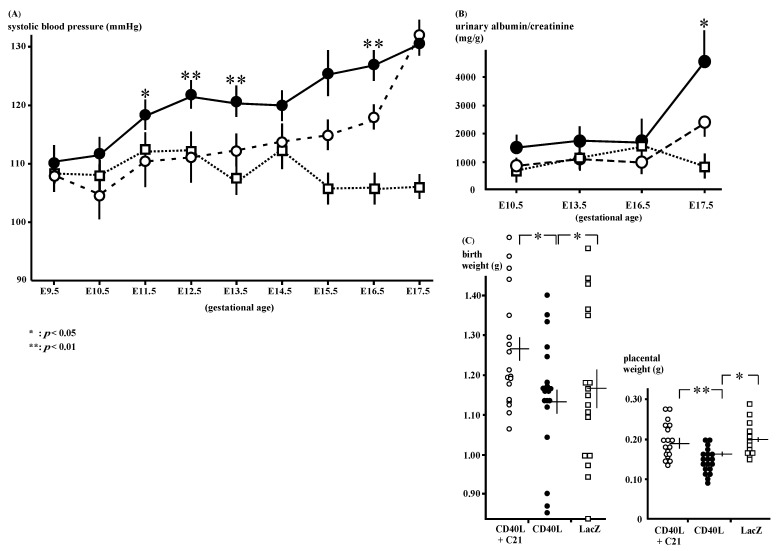
Phenotypes of CD40L and CD40L + C**21** mice. (**A**) Systolic blood pressure in CD40L mice (closed circle: *n* = 9) was increased from E12.5 onward compared to that in CD40L + C**21** mice (open circle: *n* = 9) and LacZ mice (open square: *n* = 9). (* *p* < 0.05, ** *p* < 0.01 vs. CD40L + C**21**) (**B**) Albuminuria in CD40L mice (closed circles: *n* = 10) significantly increased at E17.5 compared to LacZ mice (open squares: *n* = 5, *p* < 0.05). C**21** decreased CD40L-induced albuminuria (*n* = 10); however, it was not statistically significant. (**C**) Litter weight in CD40L + C**21** mice (open circle) was significantly higher than that of CD40L mice (closed circle) at E17.5. Placental weight in CD40L + C**21** mice (open circle) was also significantly higher than that of CD40L mice (closed circle). Data are expressed as means ± SE (* *p* < 0.05, ** *p* < 0.01).

The SBP of the CD40L mice (*n* = 9) was increased after E11.5 and C**21** (*n* = 9) significantly decreased the mice’s SBP (Figure 1A); 120 ± 2 and 110 ± 4 mmHg at E11.5 (*p* < 0.05), 123 ± 3 and 110 ± 2 mmHg at E12.5 (*p* < 0.01), 128 ± 3 and 117 ± 1 mmHg at E16.5 (*p* < 0.01), respectively.

The CD40L mice exhibited increased gestational albuminuria at E17.5 (4091.3 ± 1007.2 mg/g, *n* = 10) compared to LacZ (974.8 ± 237.5 mg/g, *n* = 5, *p* < 0.05). C**21** reduced this albuminuria; however, the decrease in the albumin/creatinine ratio was not statistically significant (CD40L + C**21** and CD40L mice [*n* = 10 each]; 2302.9 ± 336.9 mg/g and 4091.3 ± 1007.2 mg/g, respectively, at E17.5; Figure 1B, Supplementary Table S1).

### 3.2. Pregnancy Outcomes

The mean pup weight at birth was low in CD40L mice (1.15 ± 0.03 g [*n* = 19]) and was significantly improved by C**21** (1.28 ± 0.03 g [*n* = 18], *p* < 0.05; Figure 1C). C**21** also significantly increased the placental weight CD40L + C**21** [*n* = 18] vs. CD40L [*n* = 19], 0.20 ± 0.01 vs. 0.16 ± 0.01 g; *p* < 0.01; Figure 1C).

### 3.3. Intracellular Cytokine Profiles

**Figure 2:**
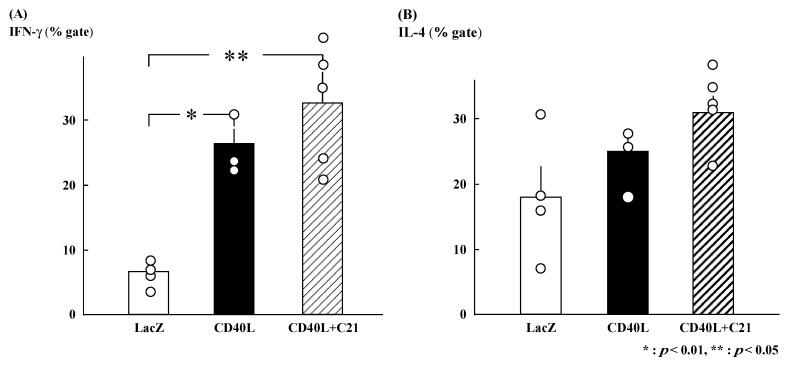
Percent intracellular cytokine-positive cells among CD4+ T cells derived from maternal spleens at E17.5. (**A**) IFN-γ-positive CD4+ T cells were significantly increased in CD40L mice (*n* = 3) compared to LacZ mice (*n* = 4). However, IFN-γ-positive CD4+ T cells in CD40L + C**21** mice (*n* = 5) were not different from CD40L mice. (**B**) The percentage of IL-4-positive CD4+ T cells was not significantly different between CD40L (*n* = 3), CD40L + C**21** (*n* = 5), and LacZ mice (*n* = 4). Data are expressed as means ± SE (* *p* < 0.01, ** *p* < 0.05).

The gated percentages of IFN-γ-positive Th cells were similar in CD40L + C**21** (*n* = 5) and CD40L (*n* = 3) CD40L mice (32.7% ± 4.7% and 26.4% ± 2.3%, respectively; Figure 2A). The gated percentages of IL-4-positive Th cells were also similar (CD40L + C**21** [*n* = 5] and CD40L [*n* = 3] mice, 30.9% ± 2.4% and 24.9% ± 3.3%, respectively; Figure 2B).

### 3.4. Flow Cytometry for B Cell Subsets Analysis

**Figure 3:**
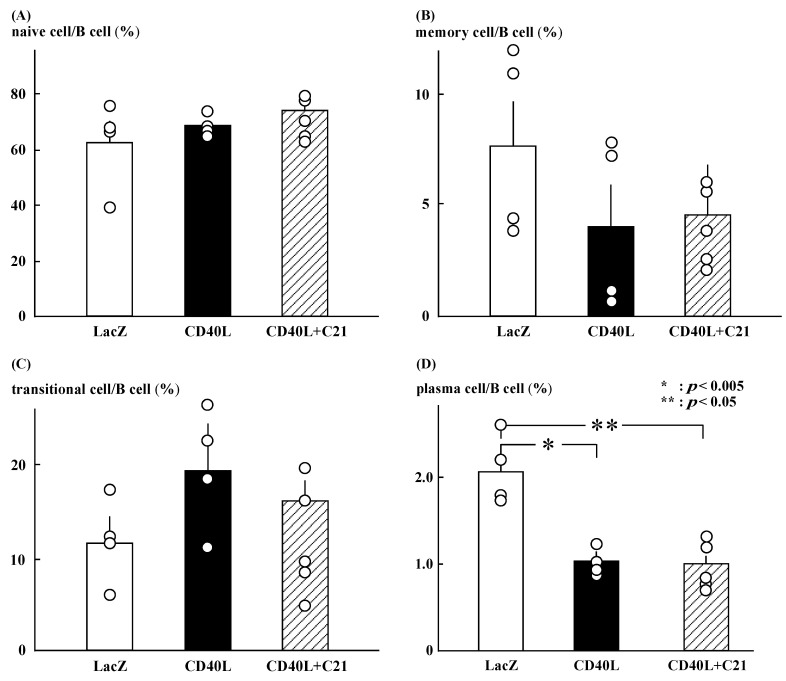
Profile of B cell subsets. (**A**–**C**) C**21** (*n* = 5) did not significantly change the ratios of naïve, memory, and transitional B cells in CD40L mice (*n* = 4). (**D**) The ratio of plasma cells was significantly lower in CD40L mice than in LacZ mice (*n* = 4) (* *p* < 0.005, ** *p* < 0.05) and was not changed by C**21**.

C**21** did not significantly change the ratios of naïve, memory, and transitional B cells in CD40L mice (Figure 3A–C). The ratio of plasma cells was significantly lower in CD40L mice than in LacZ mice (1.0 ± 0.1 [*n* = 5] vs. 2.1 ± 0.2 [*n* = 4], *p* < 0.005) and was not changed by C**21** (1.0 ± 0.1 [*n* = 5]; Figure 3D). The ratio was calculated as the number of each cell fraction to the total number of B cells.

### 3.5. Cytokine Plasma Concentrations

**Figure 4:**
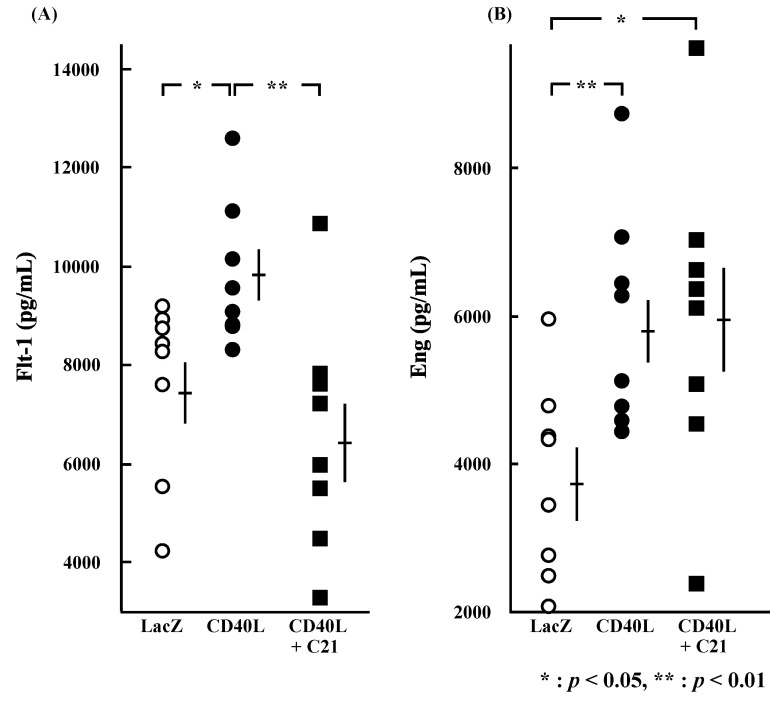
Plasma concentrations of sFlt-1 and sEng at E17.5. Plasma sFlt-1 (**A**) (*p* < 0.05) and sEng (**B**) (*p* < 0.01) concentrations were significantly greater in CD40L (*n* = 8) than LacZ (*n* = 8). Plasma sFlt-1 (**A**) concentration of CD40L was significantly decreased by C**21** treatment (*n* = 8) (*p* < 0.01); however, sEng (**B**) concentrations were not significant in CD40L + C**21** mice and in CD40L mice. Data are expressed as means ± SE (* *p* < 0.05, ** *p* < 0.01).

Plasma concentrations of sFlt-1 were significantly higher in CD40L mice than in LacZ mice (9704.4 ± 508.5 vs. 7497.1 ± 634.8 pg/mL, *p* < 0.05 [*n* = 8 each]; Figure 4A) and were significantly reduced by C**21** (6475.9 ± 829.2 pg/mL, *p* < 0.01 [*n* = 8]). Plasma concentrations of sEng were also significantly higher in CD40L mice than in LacZ mice (5909.1 ± 525.5 vs. 3768.0 ± 465.9 pg/mL, *p* < 0.01 [*n* = 8 each]); however, they were not changed by C**21** (5948.7 ± 740.8 pg/mL [*n* = 8]; Figure 4B).

## 4. Discussion

VEGF and PlGF bind to Flt-1 in vascular endothelial cells and promote angiogenesis, vasorelaxation, and thromboprophylaxis [39]. sFlt-1 inhibits these actions by binding to VEGF and placental growth factor (PlGF) in the circulating blood, resulting in vasoconstriction-induced hypertension, organ damage, and thrombocytopenia due to thrombus formation. sEng inhibits the action of transforming growth factor-β (TGF-β) by binding to endoglin expressed in vascular endothelium, thereby suppressing angiogenesis and resulting in poor placentation. In our mouse model of PE [10], plasma levels of sFlt-1 and sEng were increased and showed changes similar to those in PE patients; however, C**21** treatment decreased only sFlt-1 and had no effect on sEng. Endoglin is involved in placentation by promoting TGF-β-mediated angiogenesis, while the increase of sEng in PE placenta suppresses angiogenesis, thereby contributing to poor placentation. Since C**21** failed to suppress this effect, it is possible that AngII is not involved in the pathogenesis of PE through the endoglin-TGF-β system. On the other hand, AngII may be involved in poor placentation caused by disturbed angiogenesis due to the sFlt-1-VEGF/PlGF system. Increased levels of sFlt-1 in PE may be caused by local dysregulation of the RAS, with AngII promoting sFlt-1 production via AT1R in trophoblasts [40]. Increased AngII production in the placenta and increased vascular sensitivity of AT1R to AngII are thought to promote sFlt-1 production and reduce placental angiogenesis. In a study in which rat uterine arteries were ligated to reduce uterine perfusion pressure (reduced uterine perfusion pressure [RUPP] technique), Gilbert et al. [41] found that sFlt-1 was produced by the placenta, AngII production in the placenta was increased, and the use of an AT1R antagonist decreased plasma sFlt-1 levels, suggesting that the increased sFlt-1 production induced by reduced uterine perfusion pressure was due to AT1R activation [42]. Conversely, sFlt-1 is known to cause vascular dysfunction and promote AngII action in the vascular endothelium and placenta [42].

The AngII receptor expressed in the fetus is AT2R dominant [43,44,45]. Burrell et al. [46] found that uterine arteries of pregnant sheep have higher AT2R expression in late gestation, unlike those of nonpregnant animals, which may be responsible for the decreased vascular sensitivity to AngII during pregnancy. We previously reported that serum factors in patients with PE increased the AT1R/AT2R ratio in vascular endothelial cells [31]. Since tumor necrosis factor α (TNFα) was involved in this effect, it is likely that TNFα, which is elevated in the blood of PE patients since early pregnancy, is involved in the increase of sFlt-1 by AT1R in the pathogenesis of PE [47].

The expression of these receptors from early pregnancy suggests the importance of AngII in organogenesis, particularly the growth and differentiation of the kidney, adrenal gland, heart, and liver. AT1R may activate cellular functions, leading to AngII-mediated vasoconstriction, BP elevation, renal tubular sodium absorption, and cell proliferation, and is essential for fetal development; however, it is also involved in the pathogenesis of PE due to poor placentation by mediating adverse effects such as oxidative stress, vascular endothelial dysfunction, and inflammation [48]. AT2R has opposing effects, such as AT1R inhibition, vasodilation, depressor response, and growth inhibition by apoptosis [49]. For this reason, the inhibition of the function of AT1R and AT2R can result in severe fetal damage [50]. With increased arterial pressure in response to RUPP technique-generated placental ischemia in pregnant PE model rats, the plasma VEGF concentration decreased and the plasma sFlt-1 concentration increased [41]. ARB treatment decreased the circulating level of sFlt-1 and hypertension and proteinuria caused by placental ischemia in these rats, suggesting that ARBs could improve the pathogenesis of PE by suppressing AT1R activity [51]. AT1R is involved in the pathogenesis of PE due to poor placentation by mediating adverse effects such as oxidative stress, vascular endothelial dysfunction, and inflammation [48]. AT1R also activates cellular functions, leading to AngII-mediated vasoconstriction, BP elevation, and renal tubular sodium absorption; however, it is essential for fetal development. Thus, AT1R suppression with ARBs to improve PE is a targeted approach but complete AT1R suppression is associated with impaired fetal development. In the fetus, severe renal malformations can lead to suppression of the glomerular filtration rate (GFR), resulting in decreased fetal urine output and severe amniotic fluid depletion. Administration of ARB and ACEI to pregnant women is contraindicated because they can cause ACEI/ARB fetopathy by impairing fetal development and growth [20,21,22]. AT2R has opposing effects, such as AT1R inhibition, vasodilation, depressor response, and growth inhibition by apoptosis [49]. Promoting AT2R may improve the pathogenesis of PE without severely suppressing AT1R.

In PE, one mechanism of pathogenesis is thought to be that the activation of Th1 activates B cells, which in turn secrete AT1R autoantibodies, thereby activating maternal AT1R and causing pathological conditions such as hypertension [52]. Therefore, inhibition of immune cell activity as well as inhibition of AT1R activity is an important means to suppress the pathogenesis of PE. Indeed, in our mouse model of PE, the Th1 system was activated in spleen cells, and plasma cells were activated in B cells, which promoted antibody production by B cells. Conversely, because AT1R promotes spleen cell proliferation and activates immunity, it was thought that suppressing AT1R by C**21** would ameliorate the pathogenesis of PE [53]; however, C**21** failed to suppress T cell or B cell activity in this present study. PE causes hypertension and proteinuria via poor placentation due to impaired immune tolerance for fetal components in early pregnancy. Among various immune responses, T cells were active in our PE model mouse [10], and C**21** could not suppress the activation of T cells. Furthermore, plasma cells among B cells were decreased in our mouse model; however, C**21** was not involved in this change either, suggesting that C**21** acts via AT2R to lower blood pressure without affecting these immune responses.

AT2R knockout increased the BP of pregnant mice [30,47], whereas C**21** reduced the BP in PE model mice in this study, suggesting that AT2R plays an important role in BP regulation in pregnant mice. Furthermore, AT2R blockade reduced uterine arterial blood flow [54], suggesting that AT2R also plays an important role in the maintenance of increased uterine blood flow during pregnancy. In our study, C**21** increased the placental and fetal weights. Unlike ARBs and ACEIs, C**21** does not inhibit AT1R and thus might not impair fetal organ development or differentiation or fetal growth. These findings suggest that C**21** is a candidate drug for PE prevention and/or treatment.

C**21** also acts on thromboxane receptors. In PE, thromboxane is predominant over prostacyclin in the prostaglandin-producing system, promoting vasoconstriction and platelet aggregation, which can lead to hypertension and placental infarction. Fredgart et al. reported that C**21** inhibits its action by binding to the thromboxane receptor and acts on vasorelaxation and platelet aggregation inhibition [55]. This evidence clearly shows that C**21** has the potential to improve the course of PE by stimulating AT2R and inhibiting thromboxane.

Further effects of C**21** will undoubtedly be discovered in the future, as was the case with the recent finding of thromboxane receptor suppression in the AT2R-stimulating drug C**21**. It is clear that stimulation of AT2R in this study did not affect T cell or B cell profiles. Therefore, C**21** does not affect the primary pathology in the first stage. However, it does have an AT2R-dependent antihypertensive effect with vasorelaxation and a thromboxane receptor-dependent placental circulatory improvement effect with platelet aggregation inhibition in the second stage, which is the completed pathology, namely hypertension and hypercoagulability. The pathogenesis of PE is improved by AT2R-dependent antihypertensive action associated with vasorelaxation and thromboxane receptor-dependent action associated with inhibition of platelet aggregation to improve placental circulation. However, it is possible that antihypertensive treatment alone improves the disease state of PE. Further investigation of the effects of various antihypertensive drugs on the disease state of PE should be conducted in the future.

In this study, we used our originally developed PE mouse model. Using a mouse model allows us to obtain stable data and evaluate pathological conditions at different times during pregnancy. However, this is a mouse study, and it is difficult to apply it to humans themselves. As for the immune response, this study focused on the response of splenocytes specific for T cells and B cells, so a more comprehensive study may provide evidence that C**21** is involved in some immune responses. In addition, ARBs are contraindicated in pregnant women because they suppress the AT1R, causing fetal renal dysfunction and lethal damage to the fetus, whereas C**21** stimulates the AT2R, so its effect on the fetus is likely to be minimal. However, because there is limited experience worldwide with the use of C**21** in pregnant women, we do not know what effects it may have on the fetus and further data are needed.

## Data Availability

Data is contained within the article and Supplementary Materials.

## Supplementary Materials

The following supporting information can be downloaded at: https://www.mdpi.com/article/10.3390/cimb46090579/s1.

## List of Abbreviations

AngII	angiotensin II
AT1R	angiotensin II receptor subtype 1
AT2R	angiotensin II receptor subtype 2
C**21**	compound **21**
ICR	imprinting control region
PE	preeclampsia
sFlt-1	soluble fms-like tyrosine kinase 1
sEng	soluble endoglin
CD40	cluster of differentiation 40
CD40L	CD40 ligand
TNF	tumor necrosis factor
Th	T-helper cell
RAS	renin–angiotensin system
BP	blood pressure
ARBs	angiotensin receptor blockers
ACEIs	angiotensin-converting enzyme inhibitors
B2R	bradykinin B2 receptor
IPF	idiopathic pulmonary fibrosis
FVC	forced vital capacity
LacZ	β-galactosidase
ELISA	enzyme-linked immunosorbent assay
RT	room temperature
IFN-γ	interferon-γ
IL-4	interleukin-4
VEGFR1	vascular endothelial growth factor receptor 1
ANOVAs	one-way analysis of variance
PlGF	placental growth factor
