# Supplementary material for: Stimulation of Angiotensin II Receptor Subtype 2 Reduces Preeclampsia-like Symptoms in a Mouse Model of Preeclampsia
Source: Curr Issues Mol Biol. 2024 Sep 2;46(9):9760–71. doi: 10.3390/cimb46090579 (PMC11430795; doi:10.3390/cimb46090579)

Supplementary Figure 1.

Tcell analysis LacZ-1

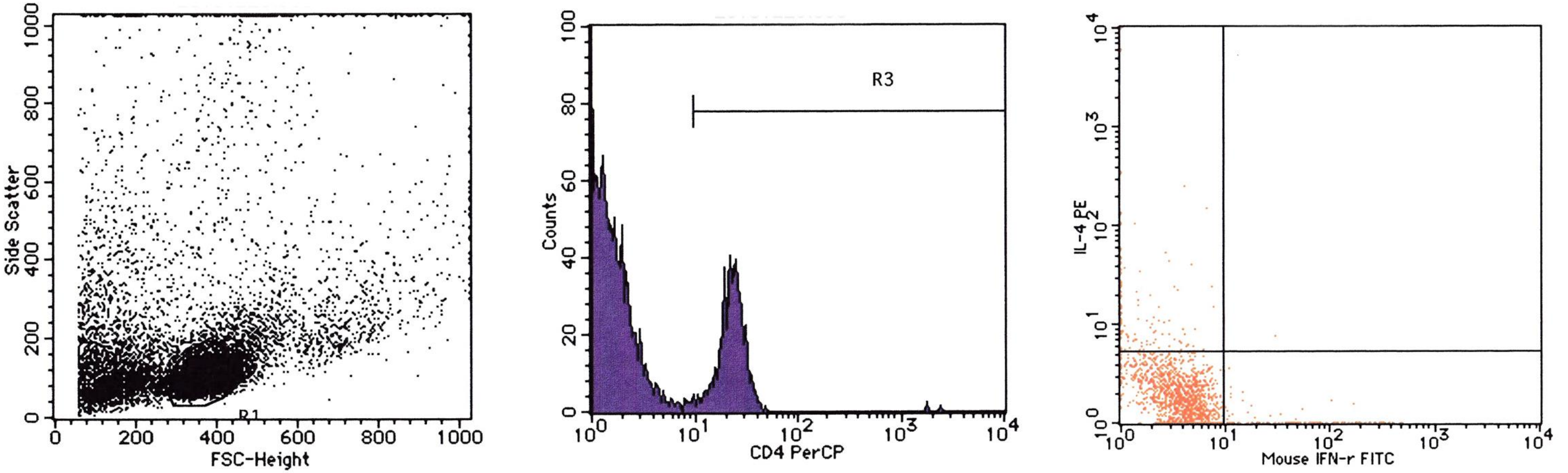

Tcell analysis LacZ-2

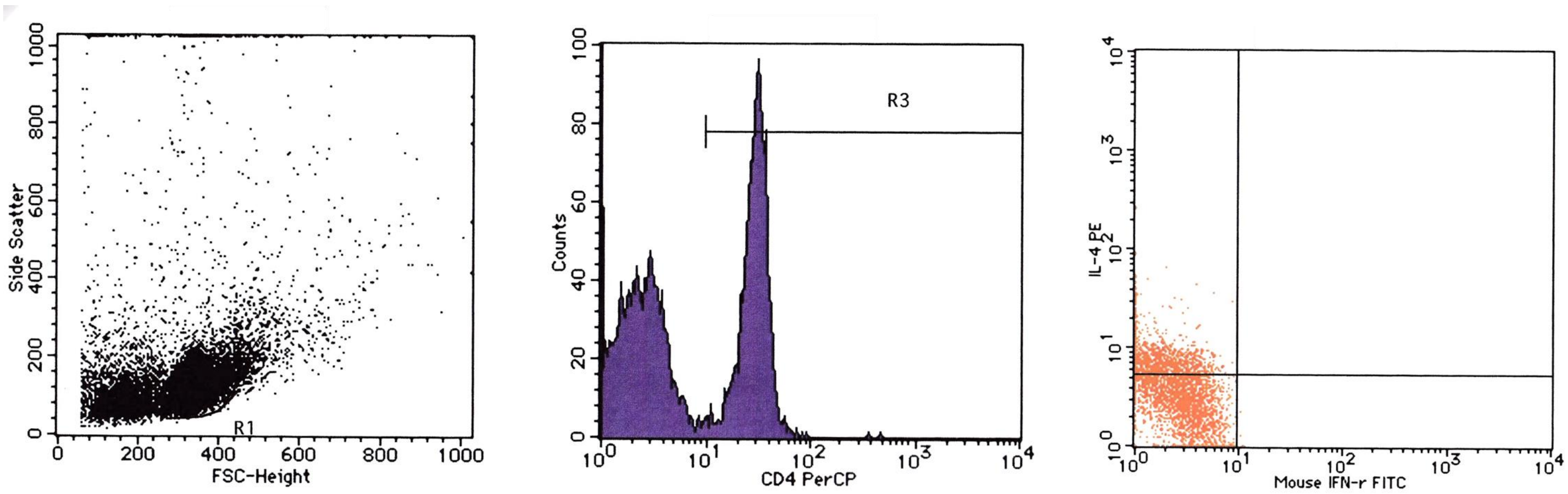

Tcell analysis LacZ-3

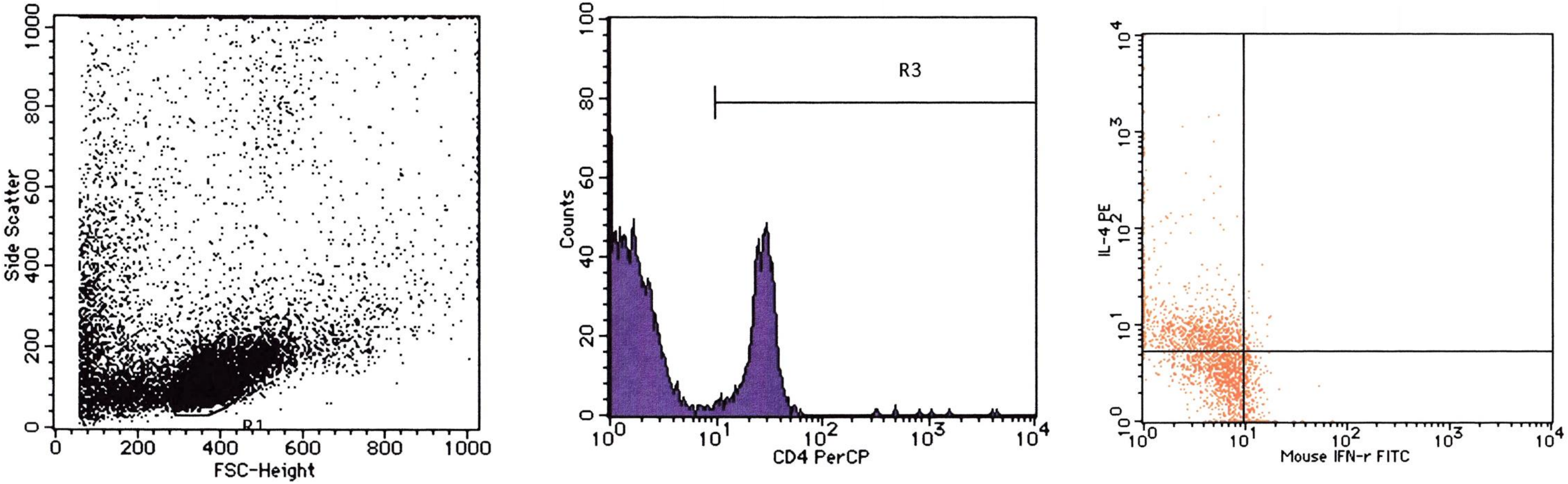

Tcell analysis LacZ-4

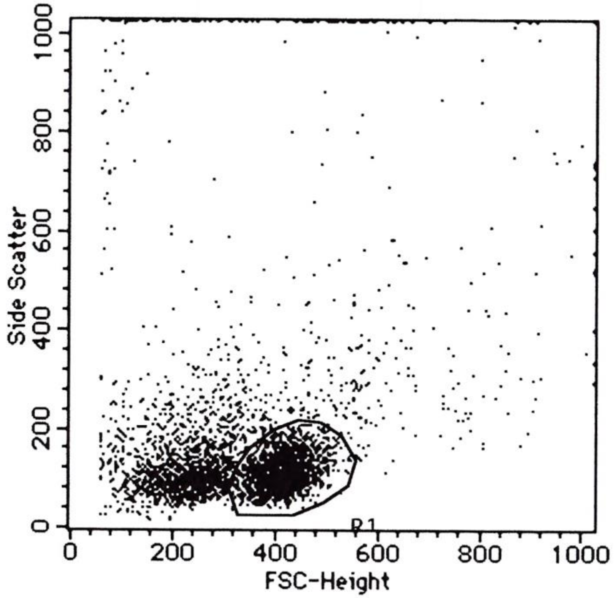

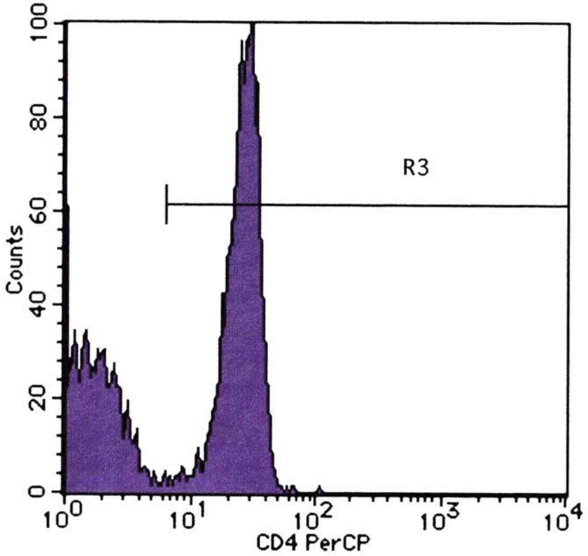

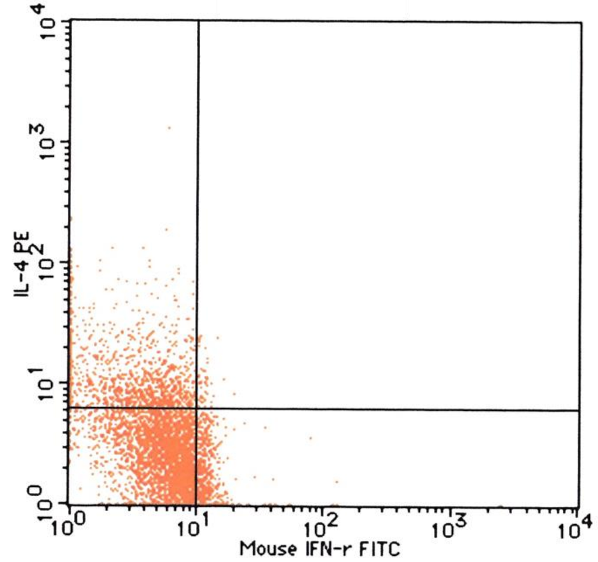

Tcell analysis CD40L-1

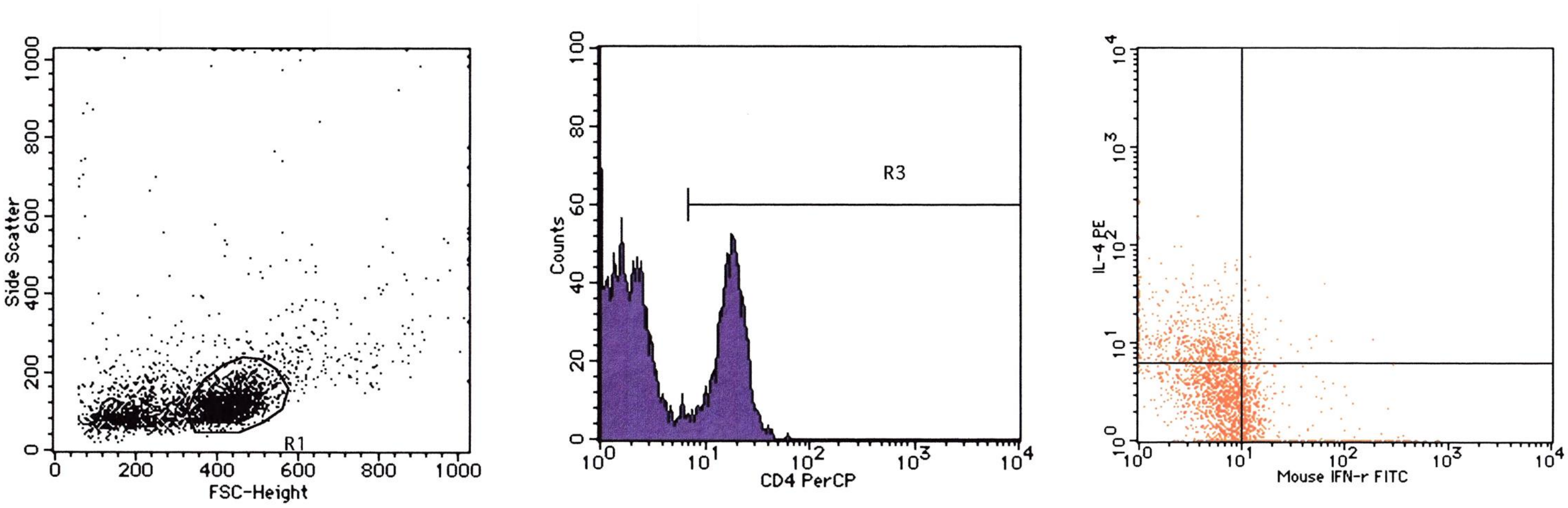

Tcell analysis CD40L-2

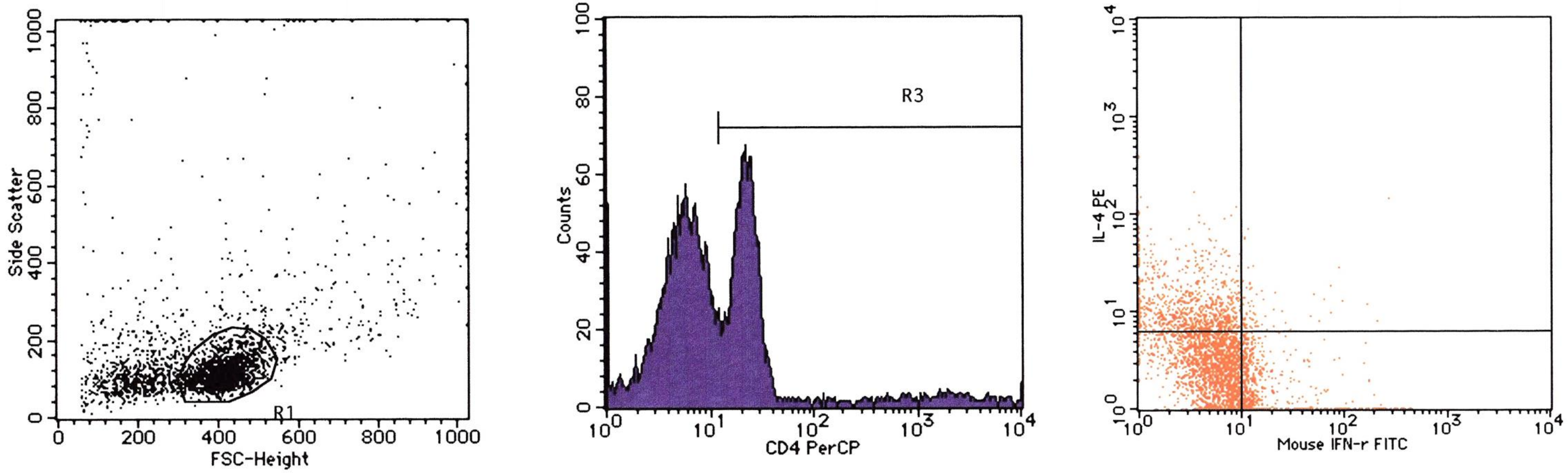

Tcell analysis CD40L-3

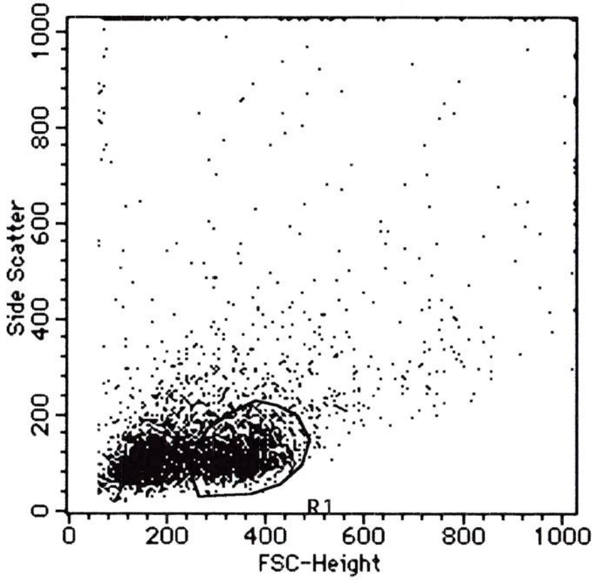

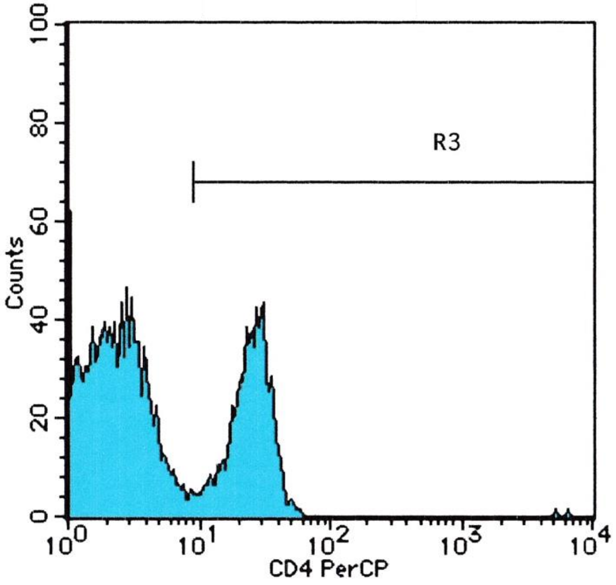

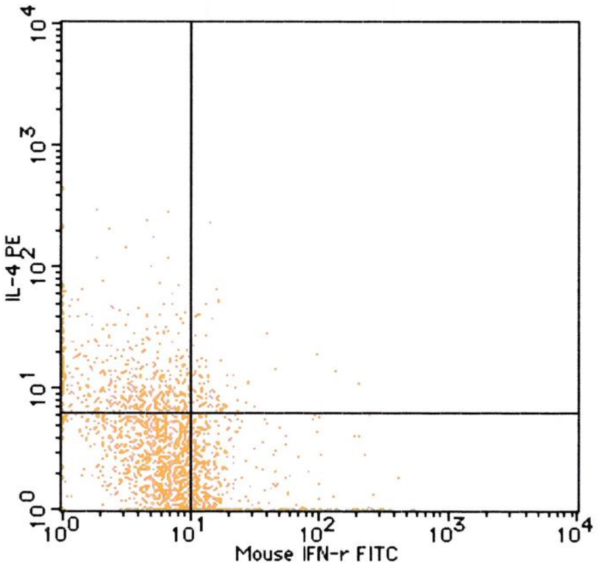

Tcell analysis CD40L with C21-1

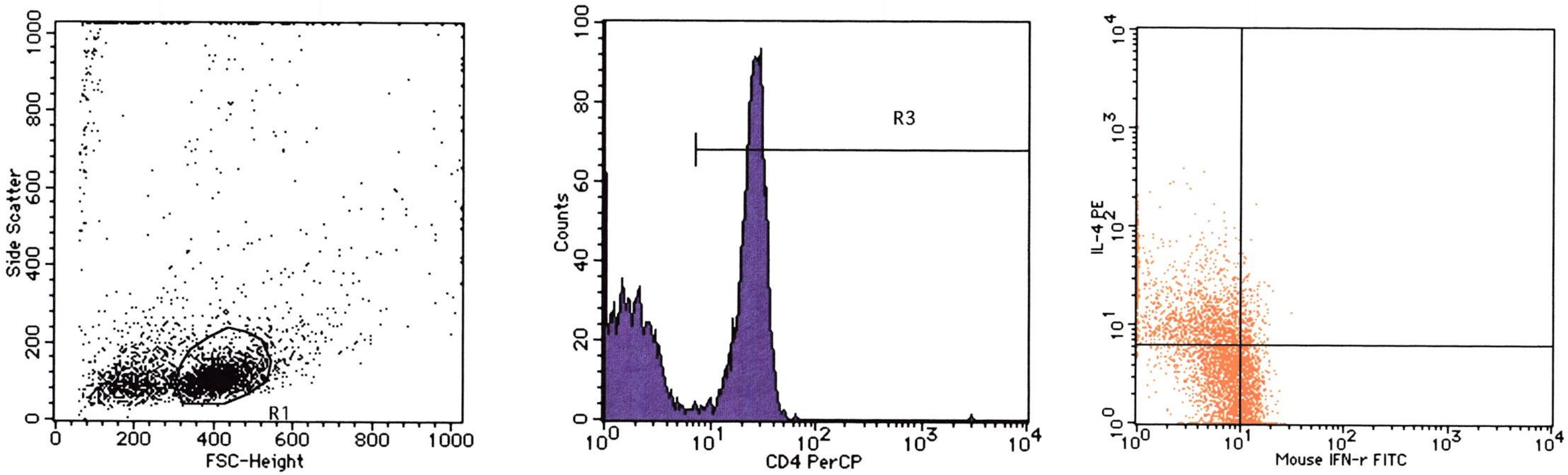

Tcell analysis CD40L with C21-2

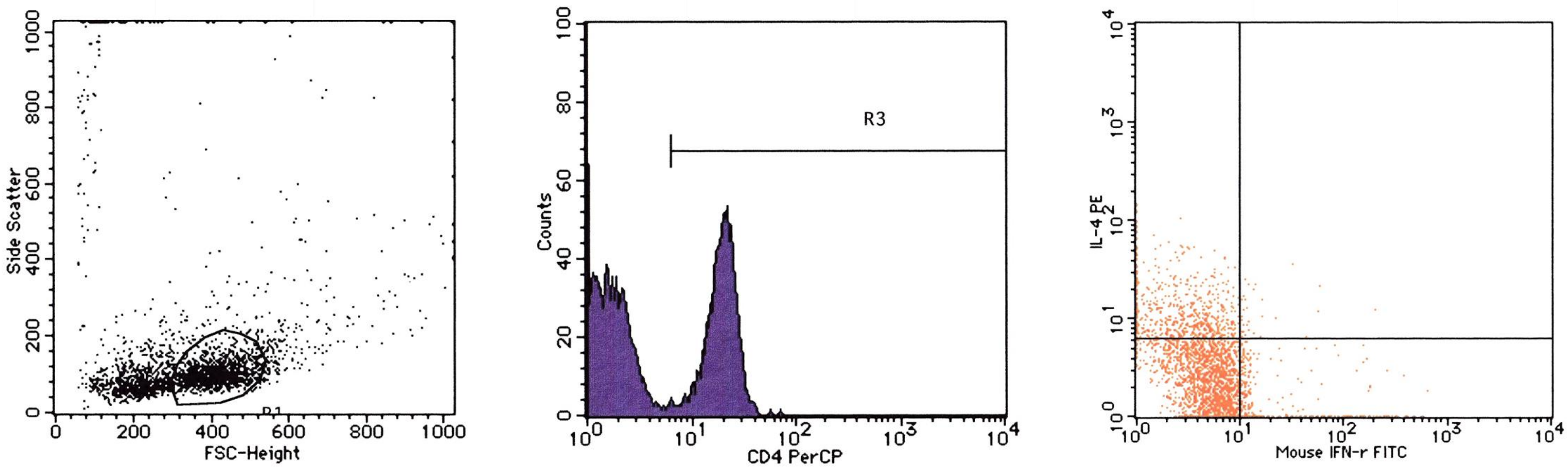

Tcell analysis CD40L with C21-3

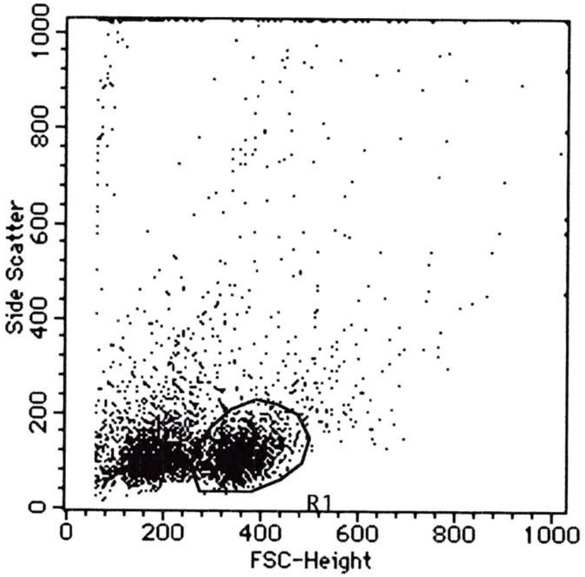

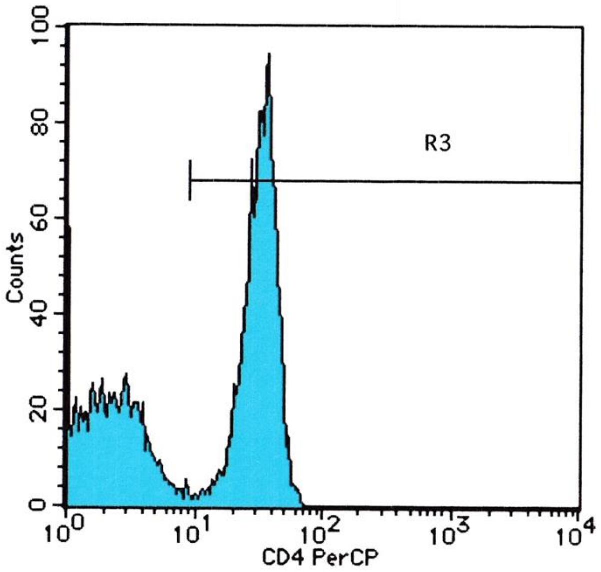

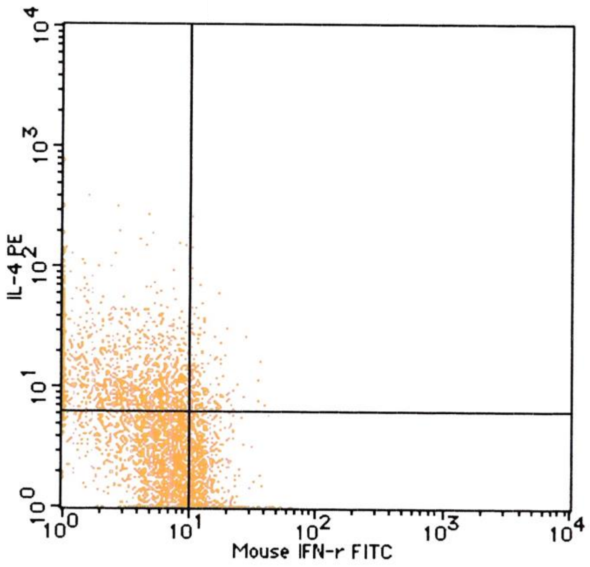

Tcell analysis CD40L with C21-4

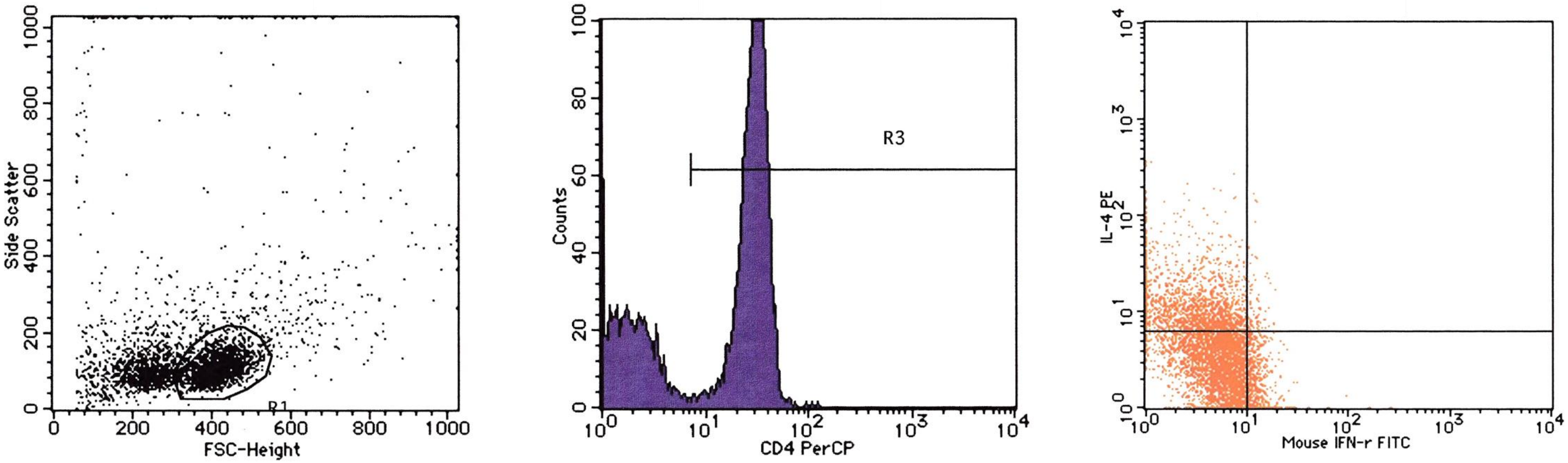

Tcell analysis CD40L with C21-5

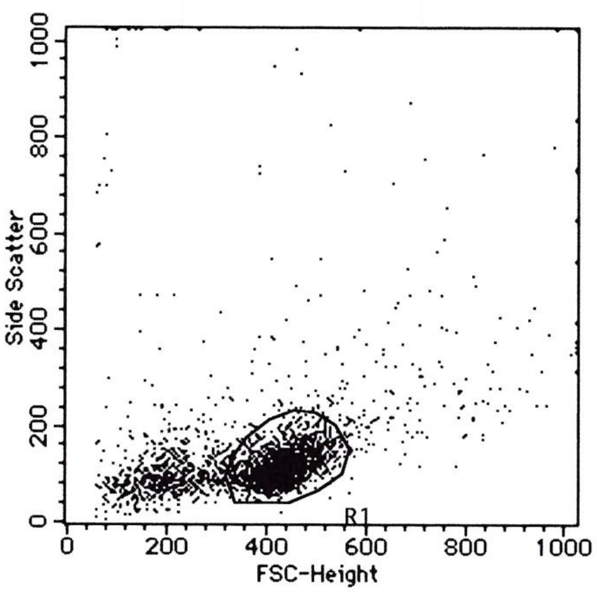

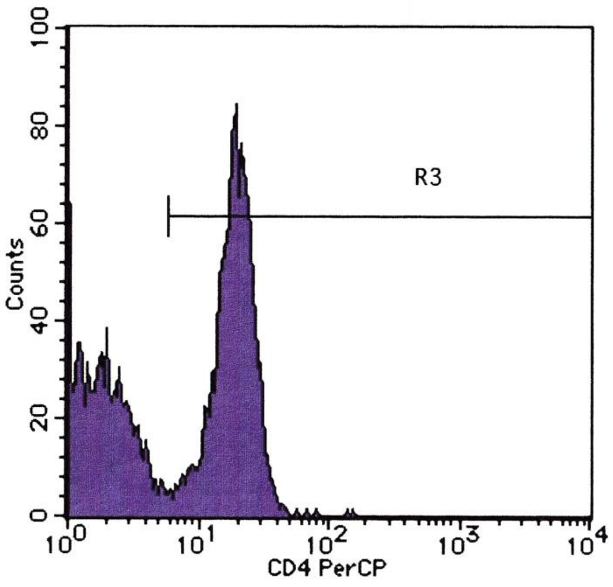

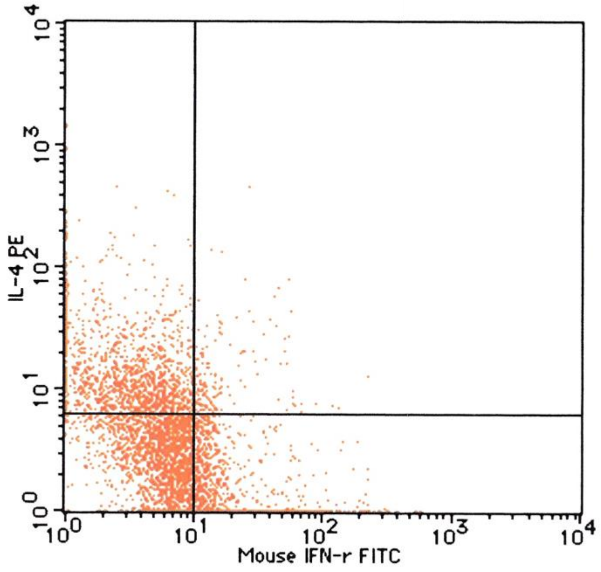

Supplement: Supplementary file 1 [file cimb-46-00579-s001.zip › Supplementary Figure S1.pdf]
