# Supplementary material for: Stimulation of Angiotensin II Receptor Subtype 2 Reduces Preeclampsia-like Symptoms in a Mouse Model of Preeclampsia
Source: Curr Issues Mol Biol. 2024 Sep 2;46(9):9760–71. doi: 10.3390/cimb46090579 (PMC11430795; doi:10.3390/cimb46090579)

Supplementary Figure 2.
Bcell analysis CD40L-1

Naïve Bcell

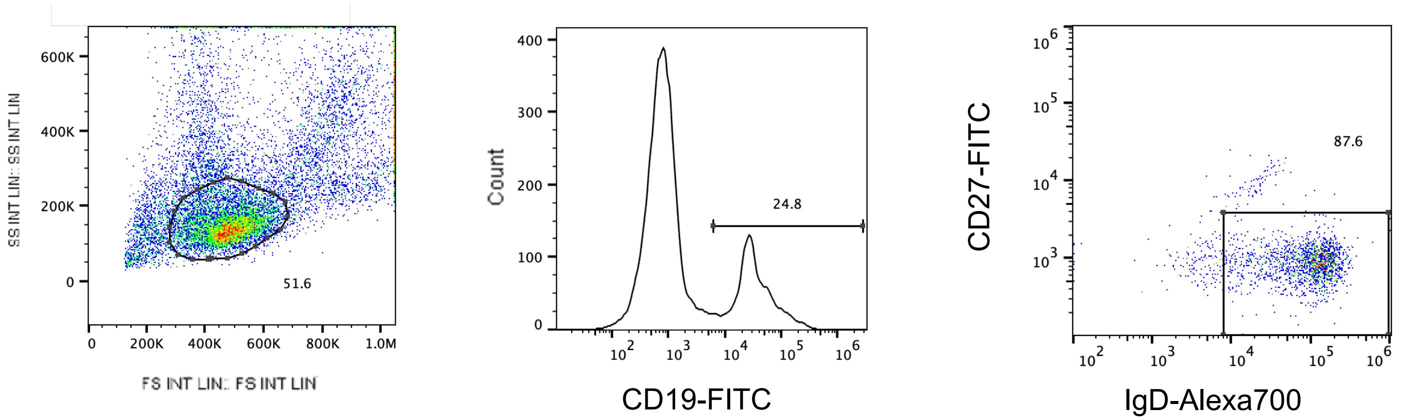

Memory Bcell

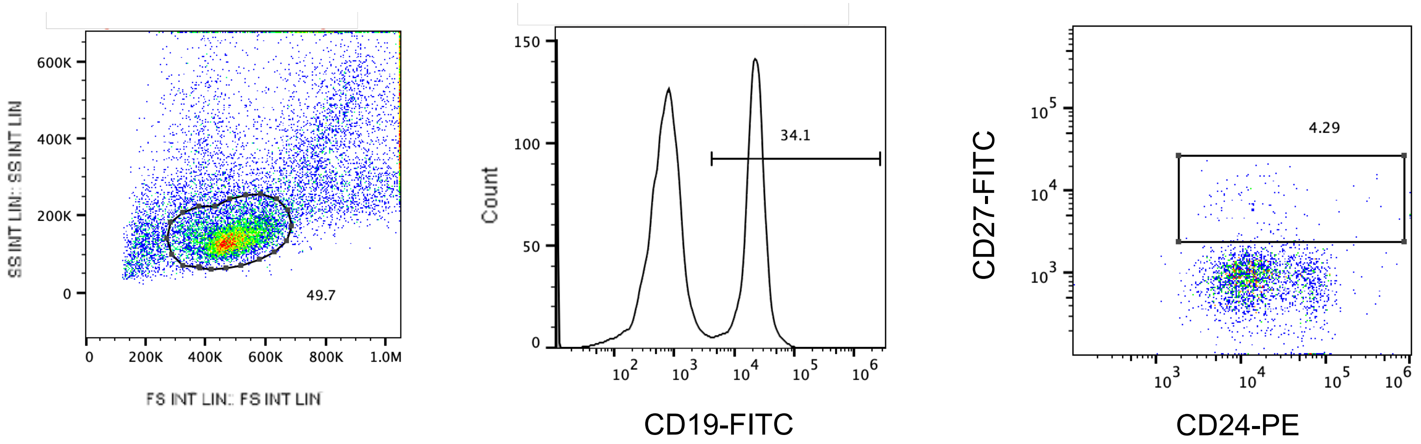

Bcell analysis CD40L-1

Transitional Bcell

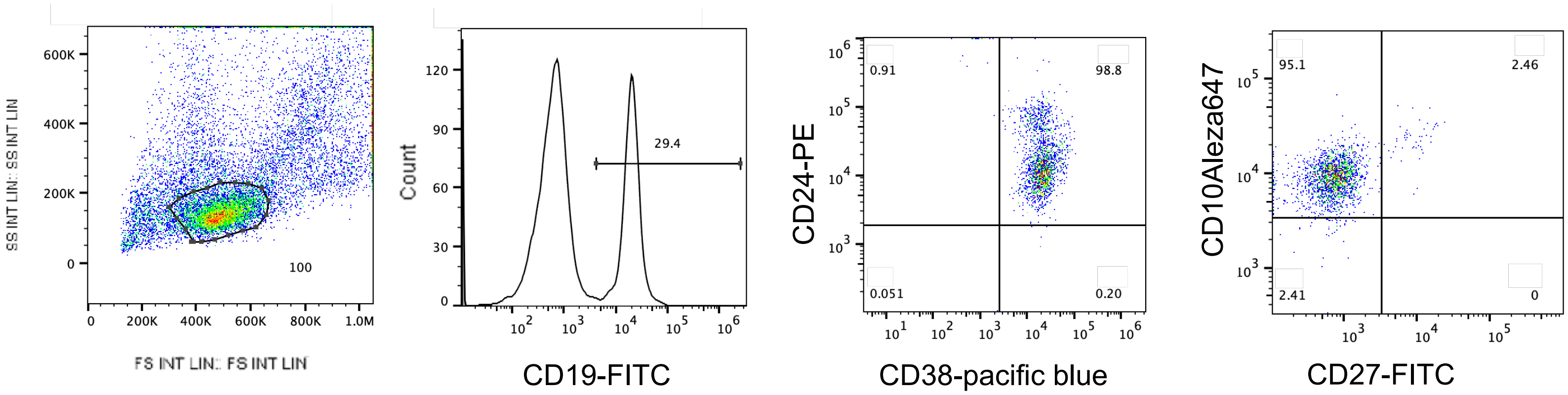

Plasmatic cell

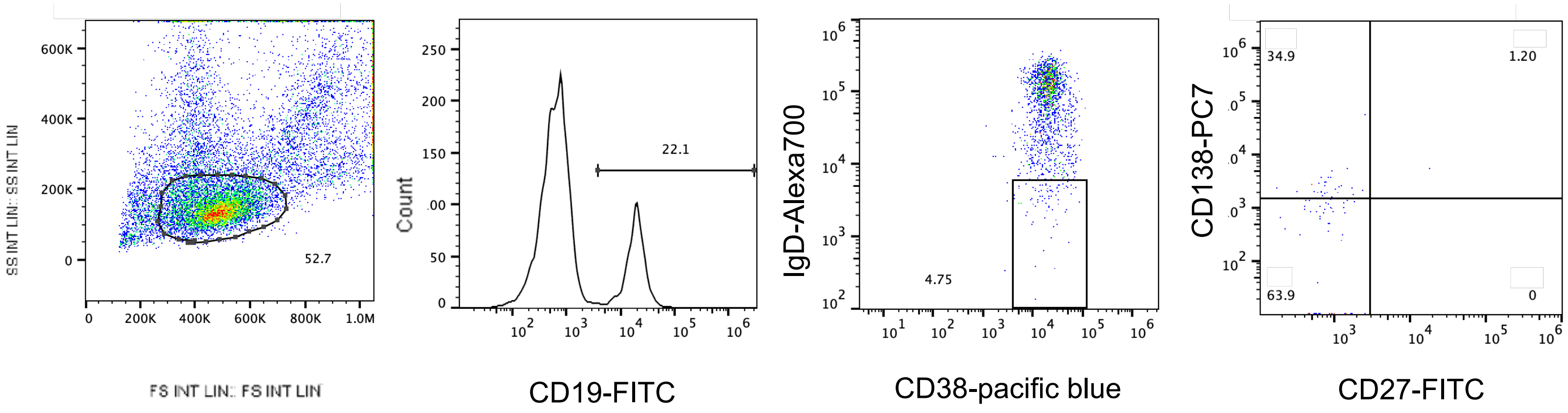

Bcell analysis CD40L-2

Naïve Bcell

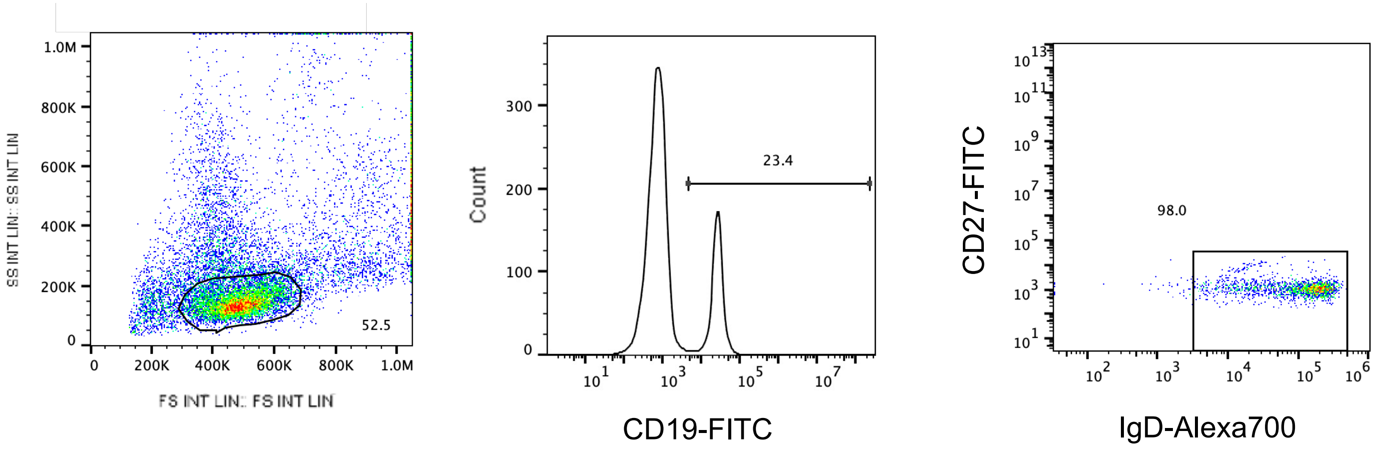

Memory Bcell

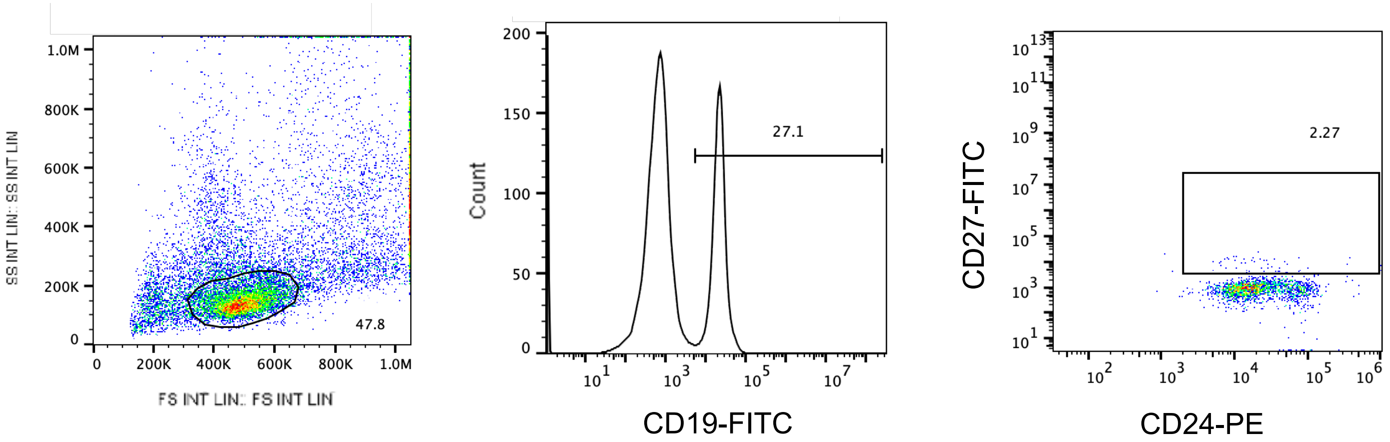

Bcell analysis CD40L-2

Transitional Bcell

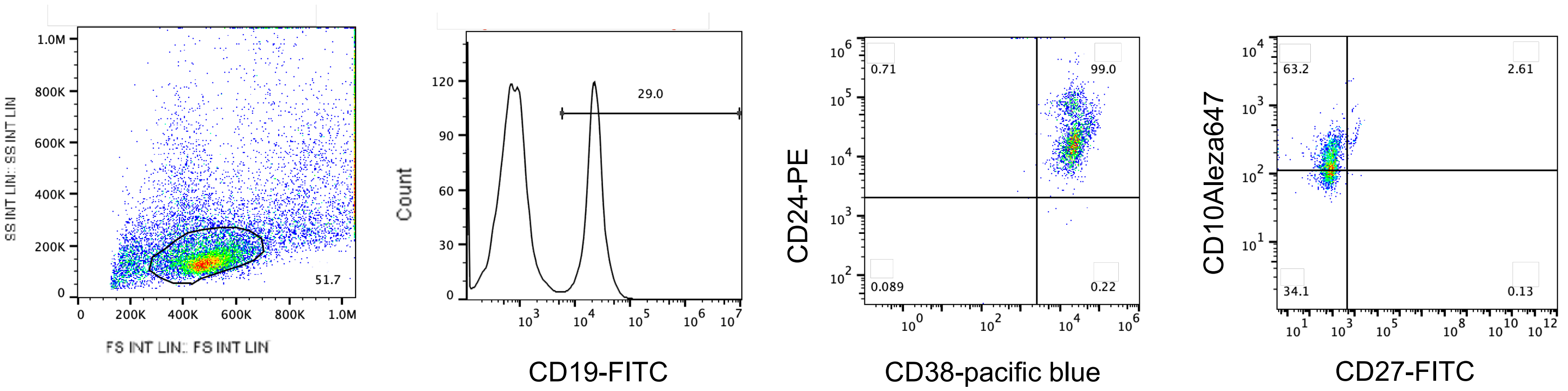

Plasmatic cell

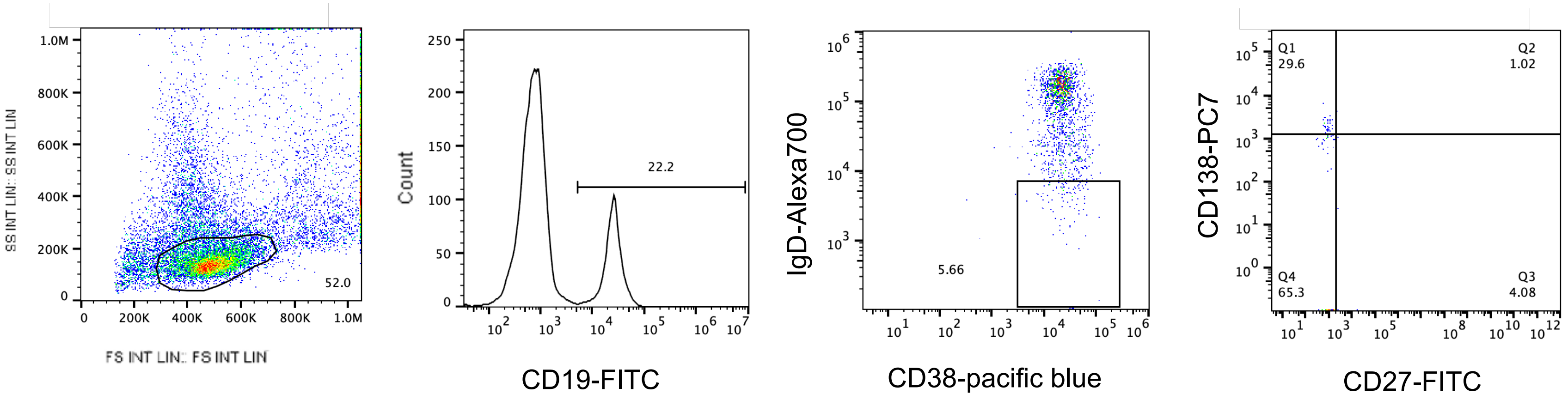

Bcell analysis CD40L-3

Naïve Bcell

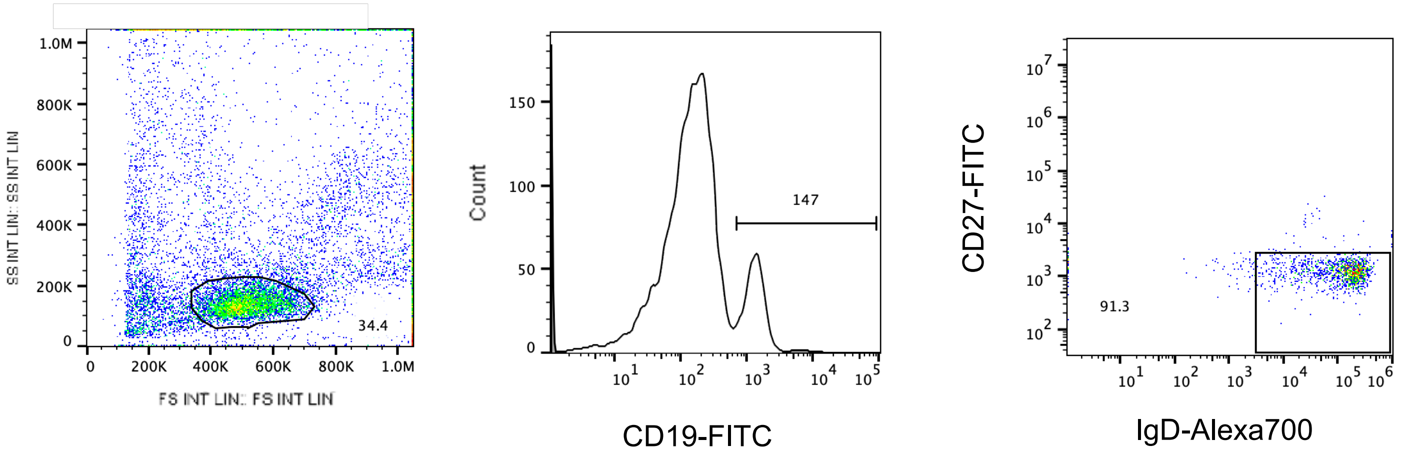

Memory Bcell

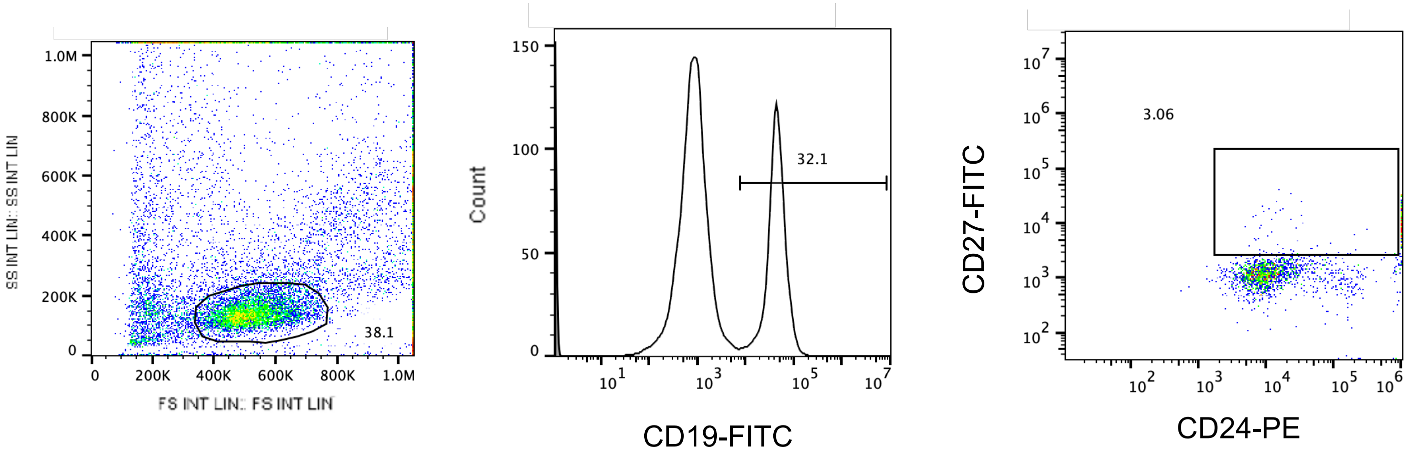

Bcell analysis CD40L-3

Transitional Bcell

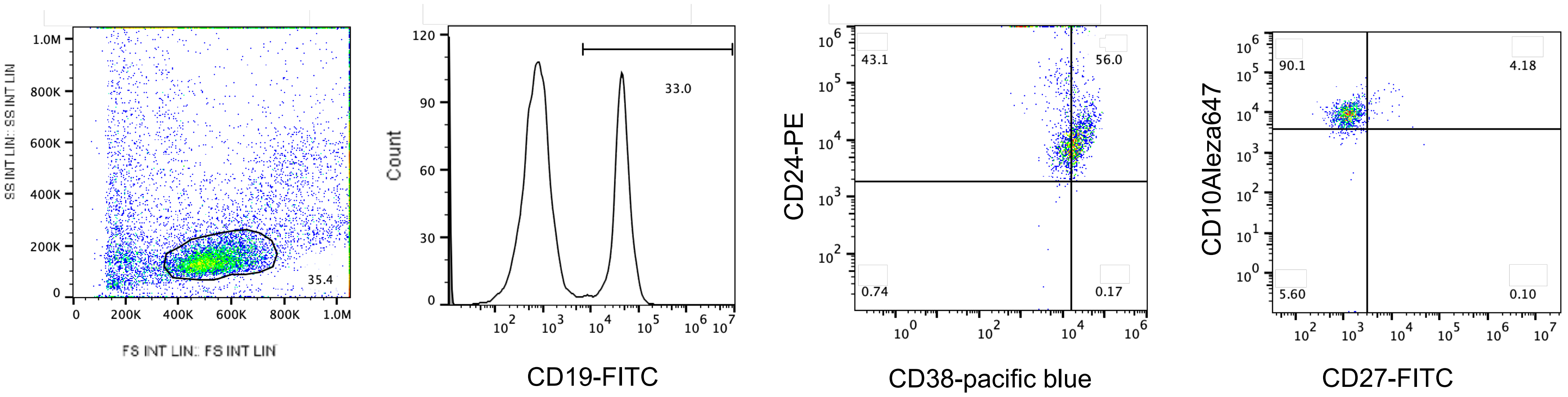

Plasmatic cell

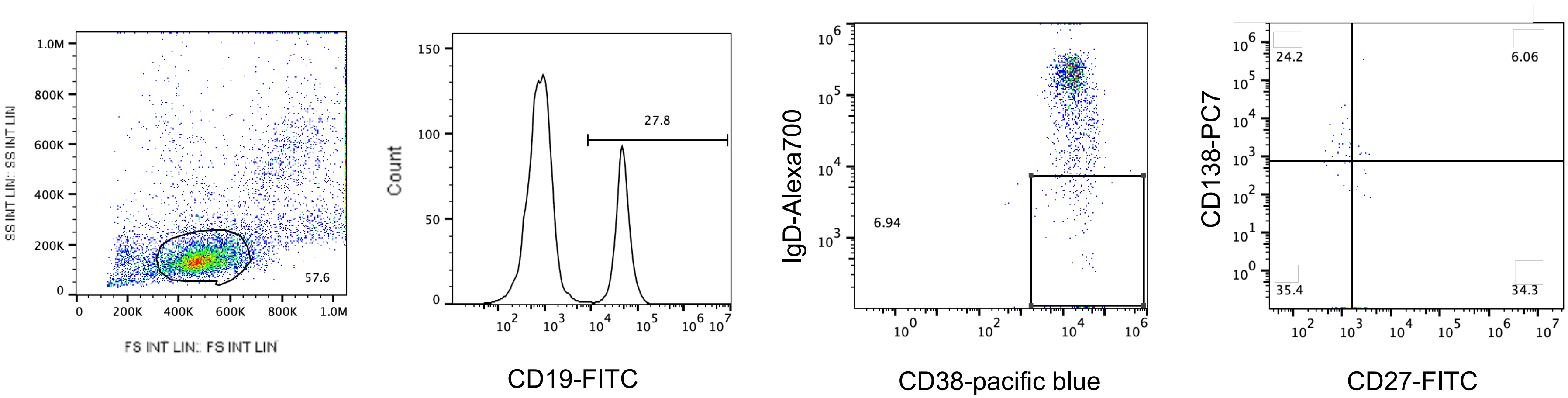

Bcell analysis CD40L-4

Naïve Bcell

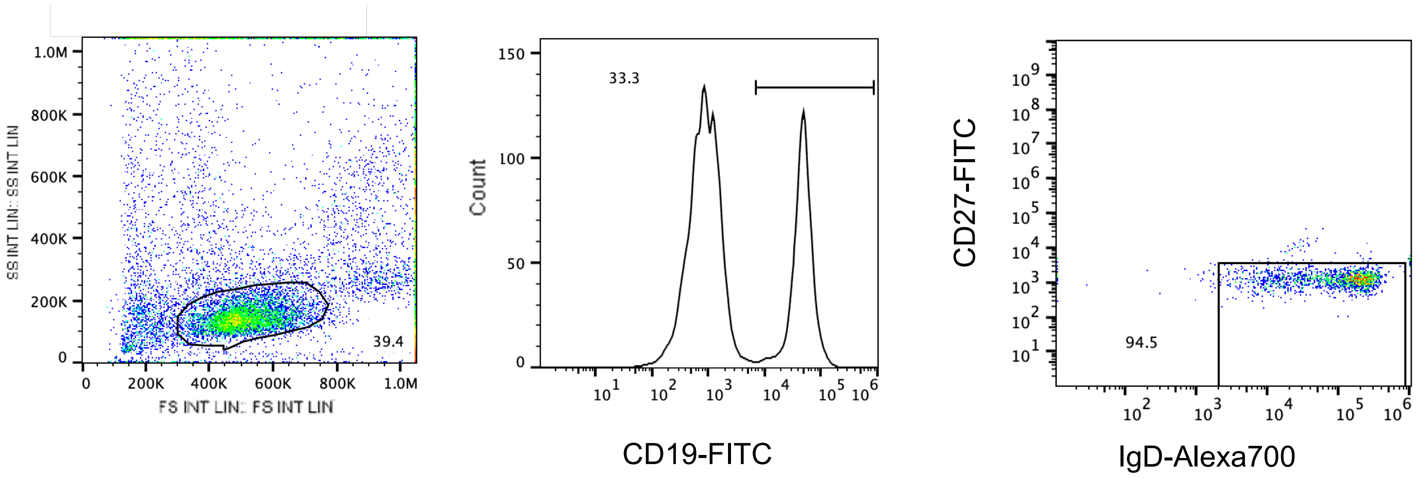

Memory Bcell

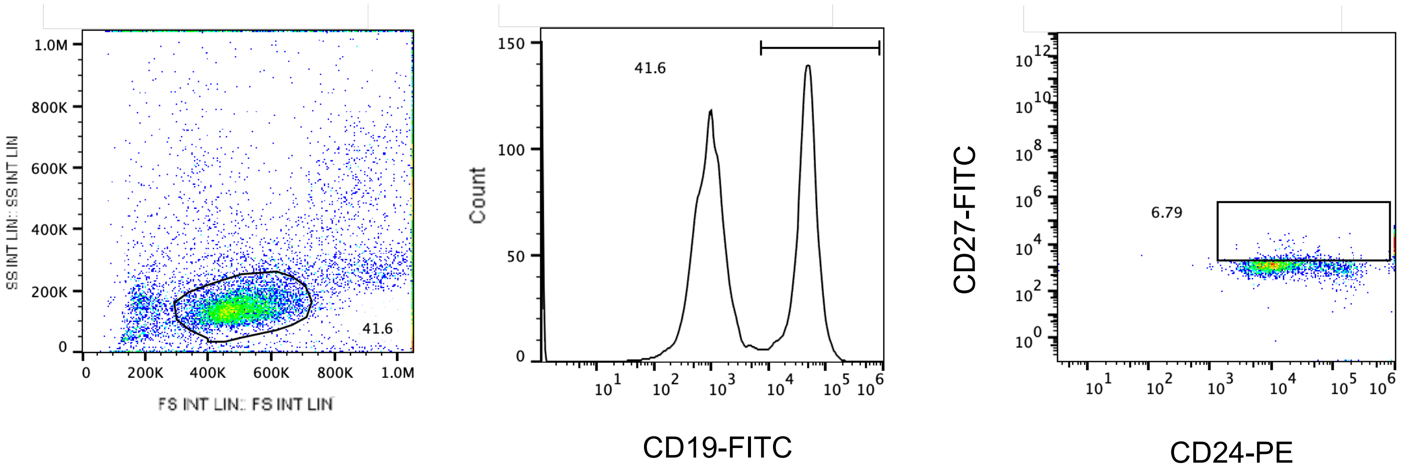

Bcell analysis CD40L-4

Transitional Bcell

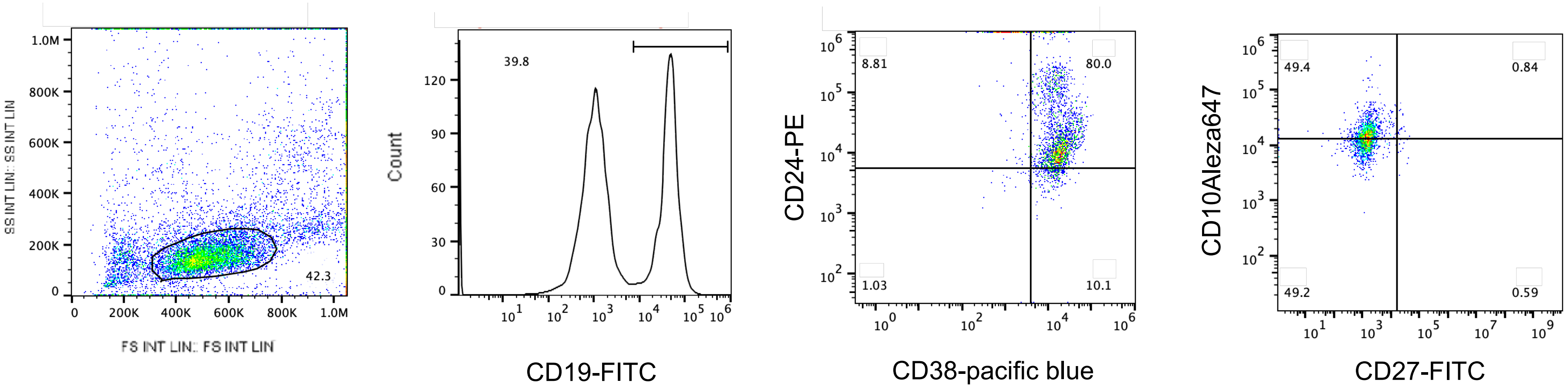

Plasmatic cell

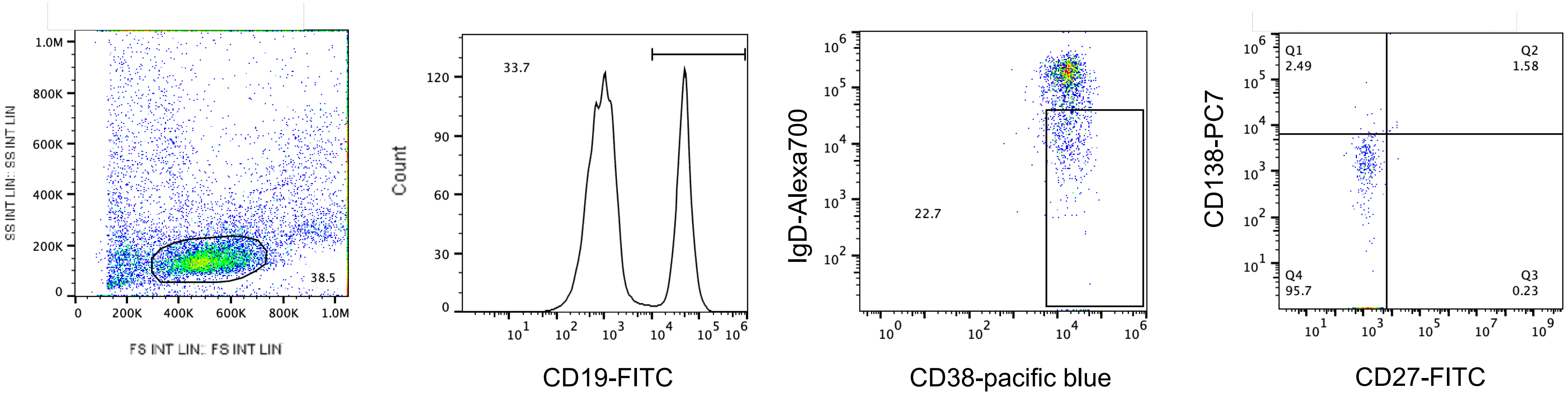

Bcell analysis CD40L with C21-1

Naïve Bcell

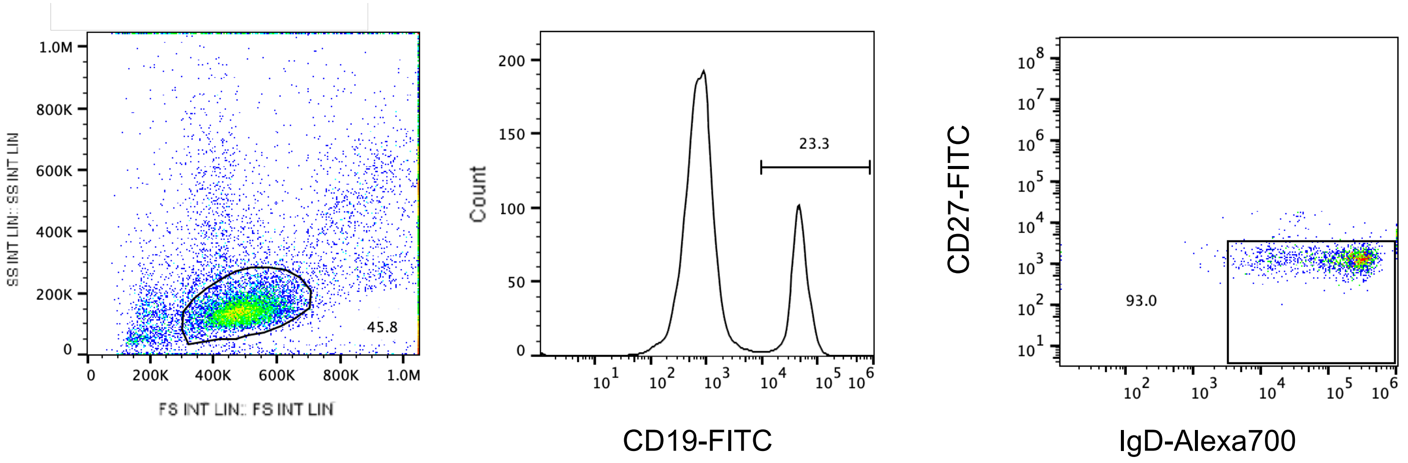

Memory Bcell

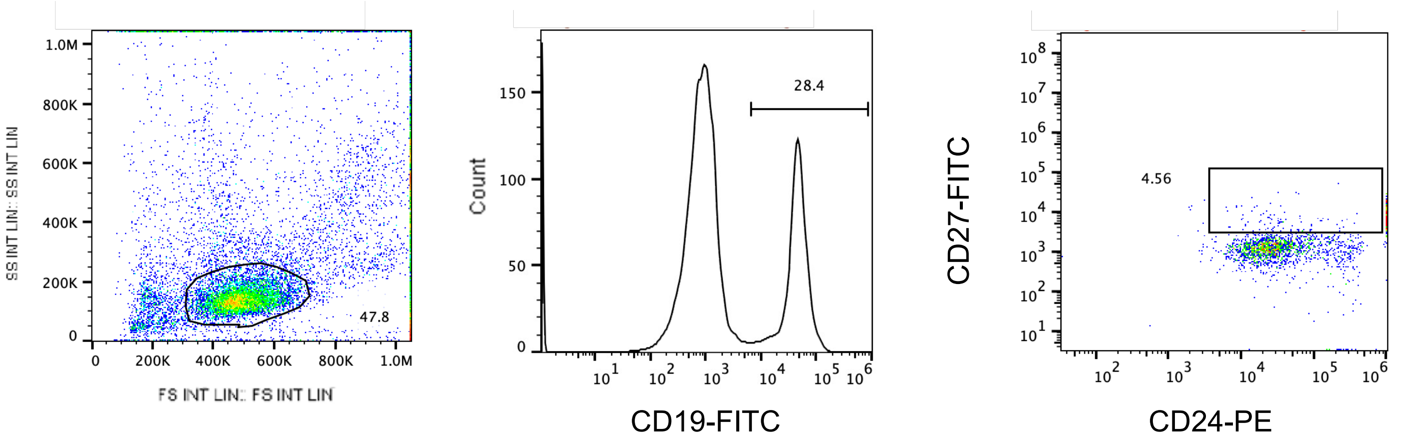

Bcell analysis CD40L with C21-1

Transitional Bcell

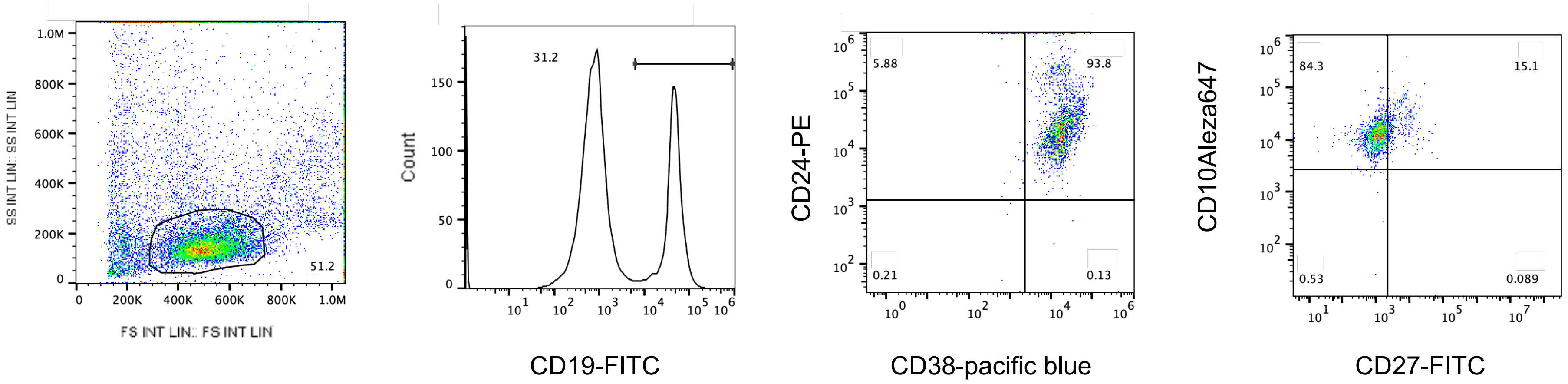

Plasmatic cell

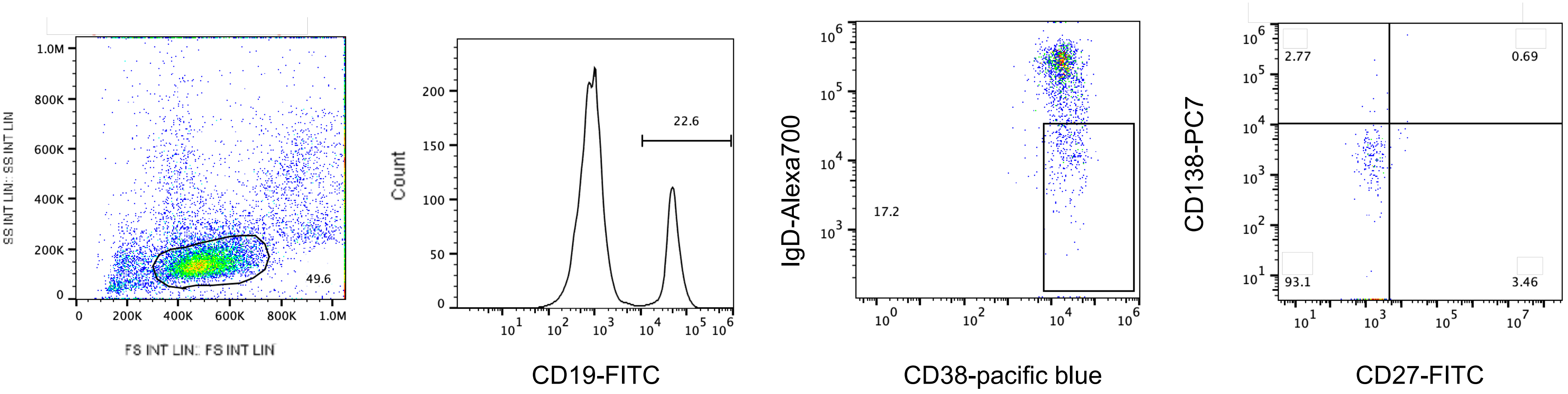

Bcell analysis CD40L with C21-2

Naïve Bcell

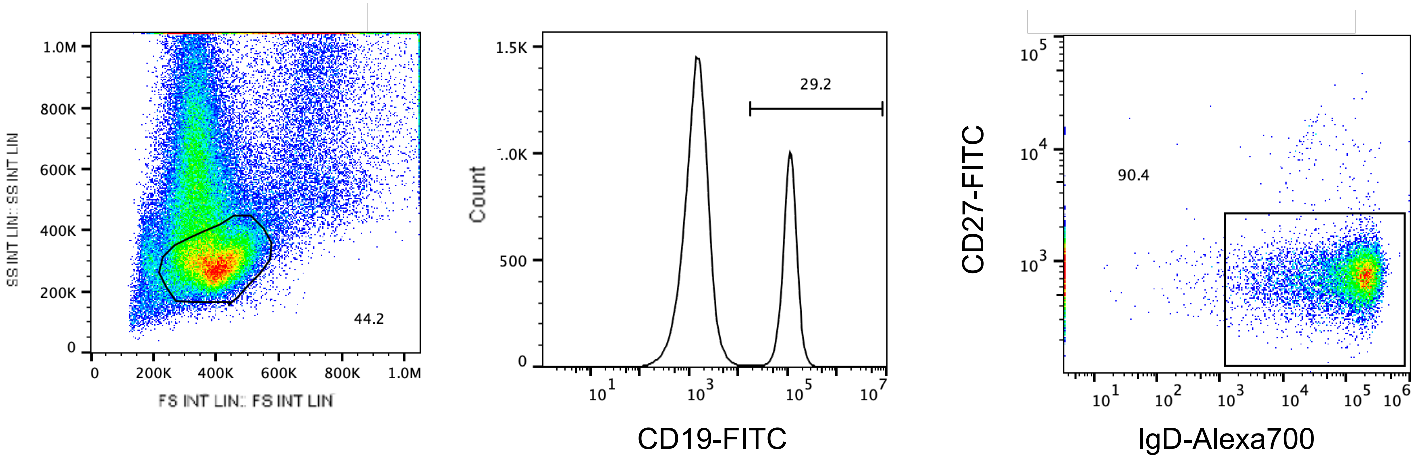

Memory Bcell

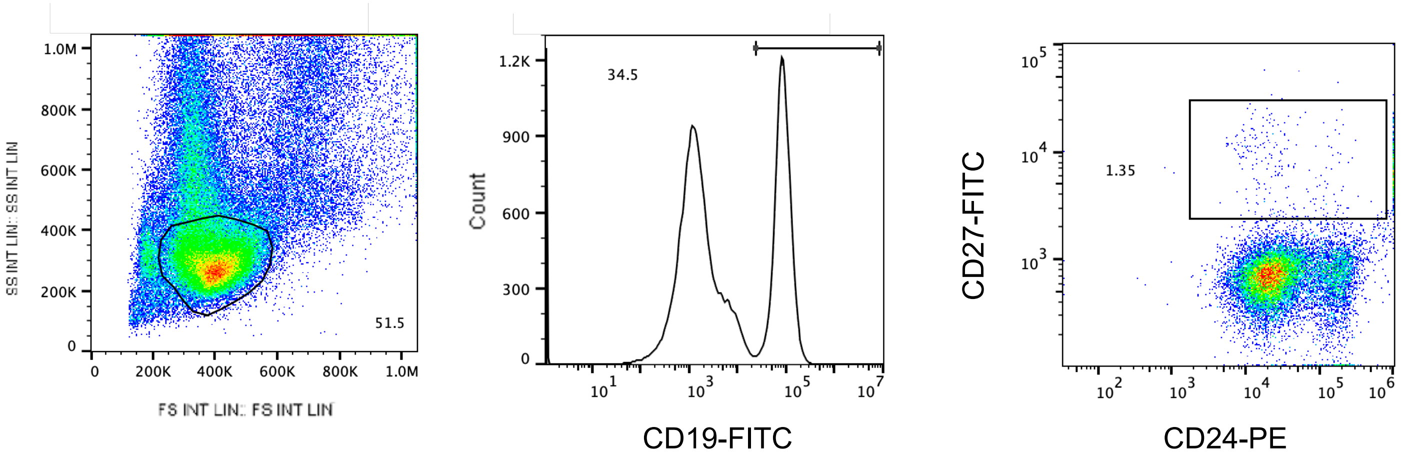

Bcell analysis CD40L with C21-2

Transitional Bcell

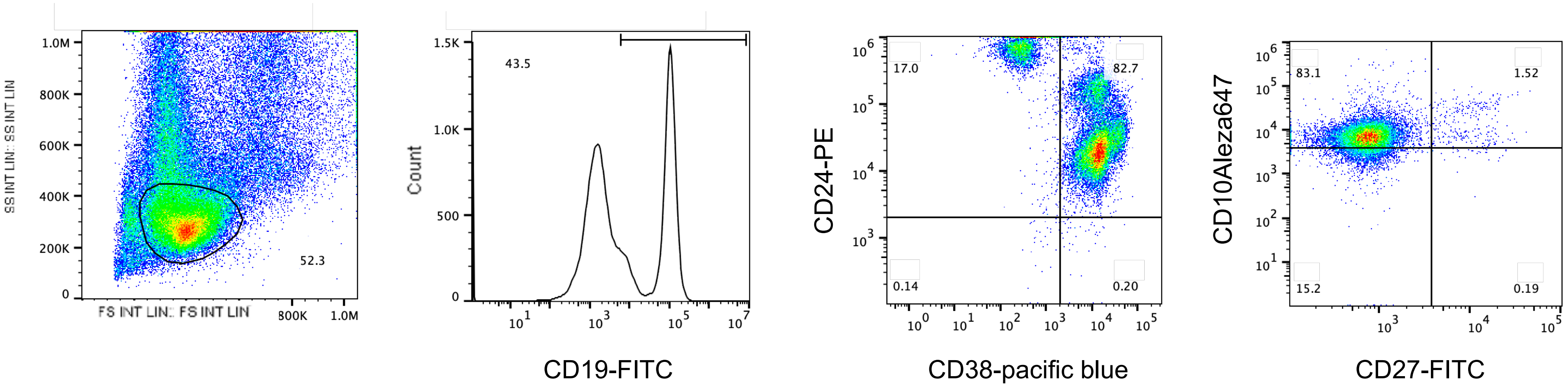

Plasmatic cell

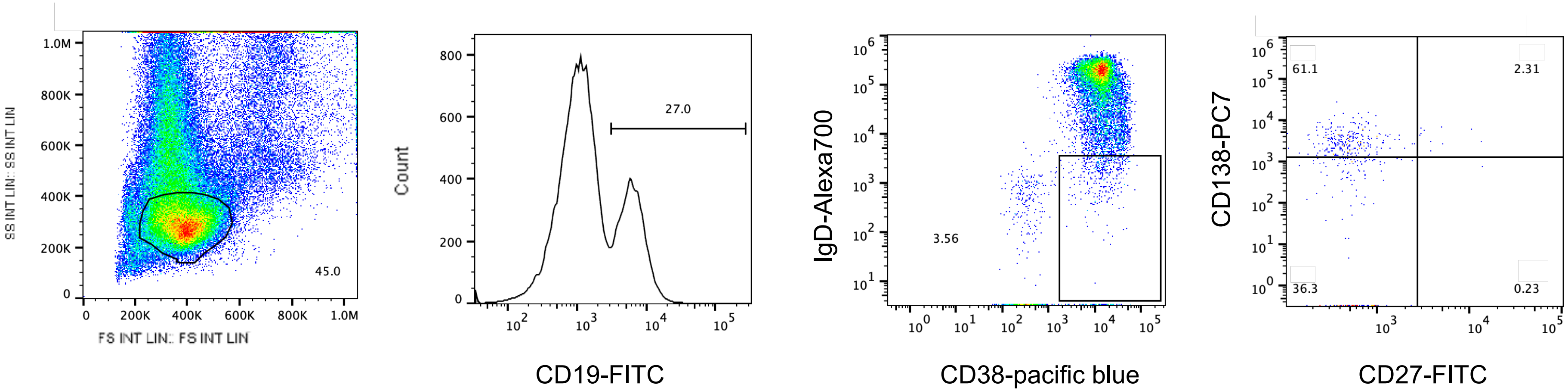

Bcell analysis CD40L with C21-3

Naïve Bcell

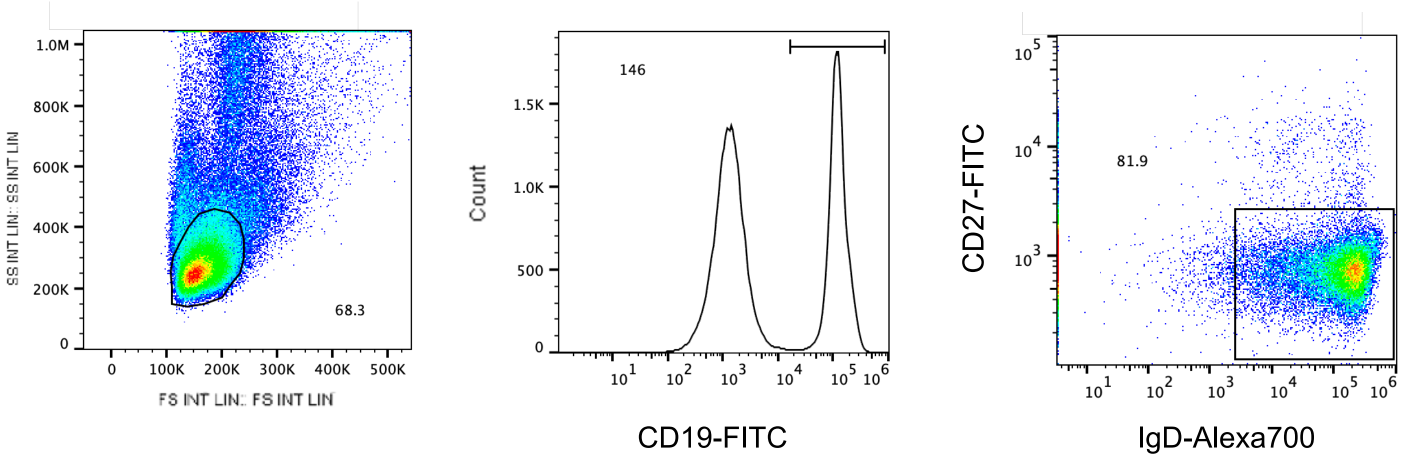

Memory Bcell

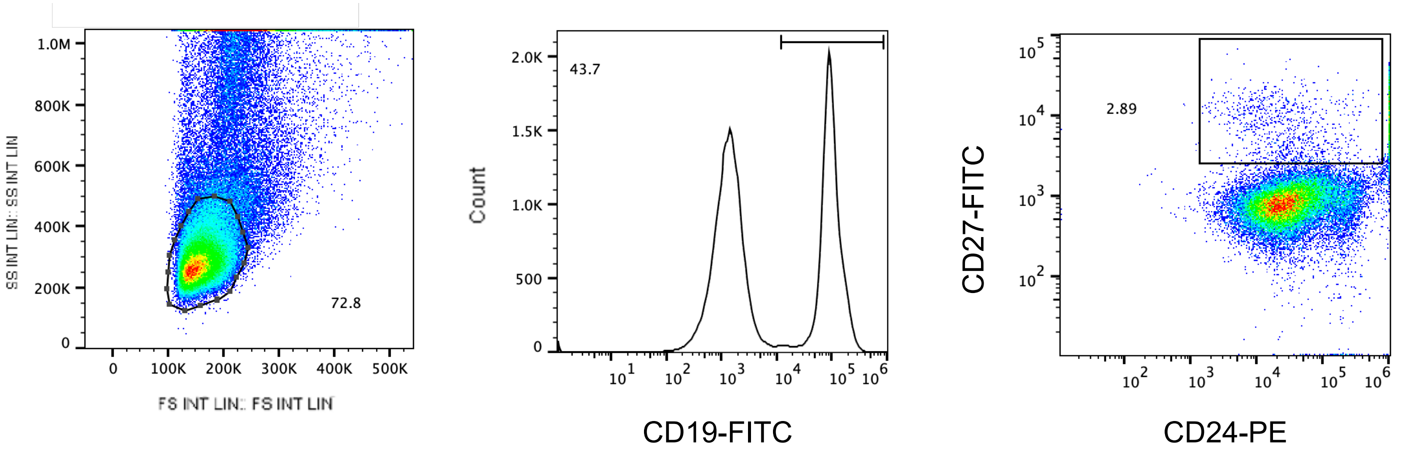

Bcell analysis CD40L with C21-3

Transitional Bcell

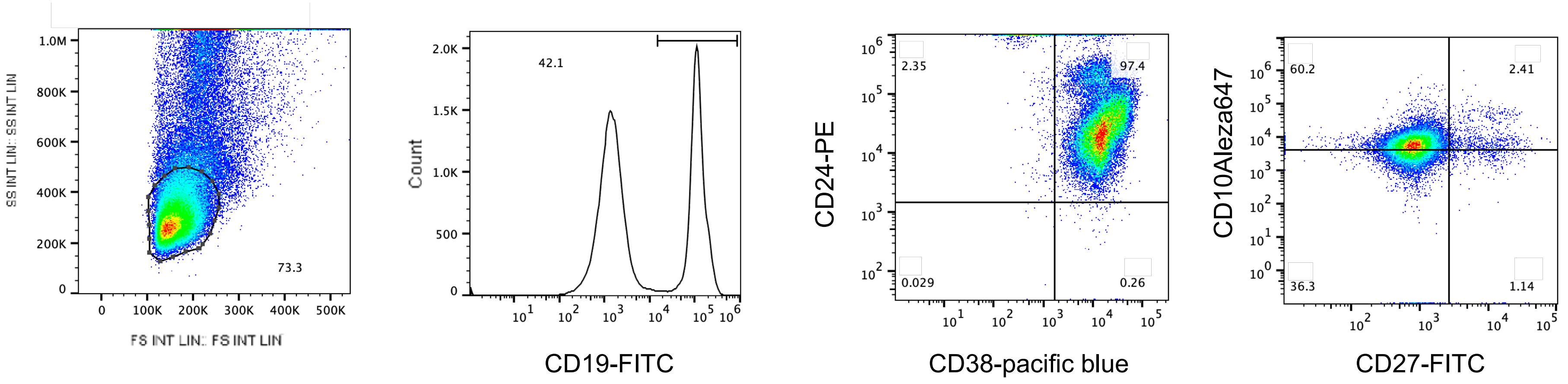

Plasmatic cell

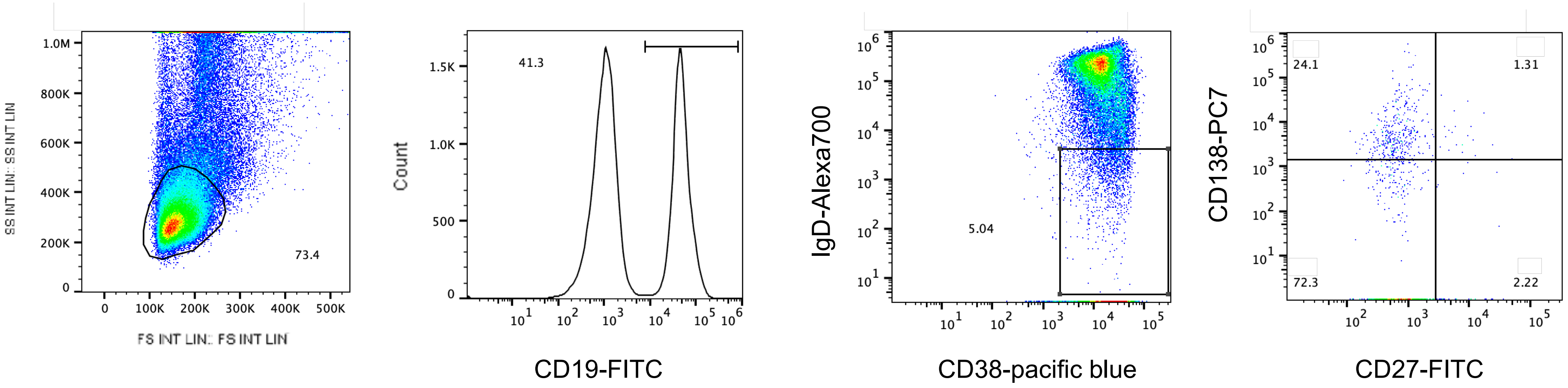

Bcell analysis CD40L with C21-4

Naïve Bcell

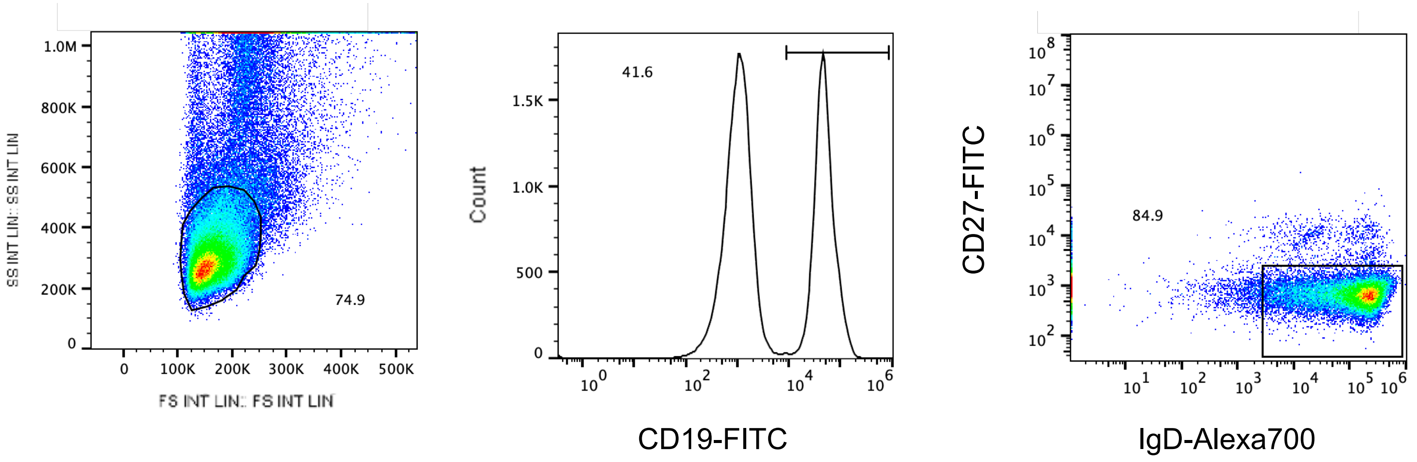

Memory Bcell

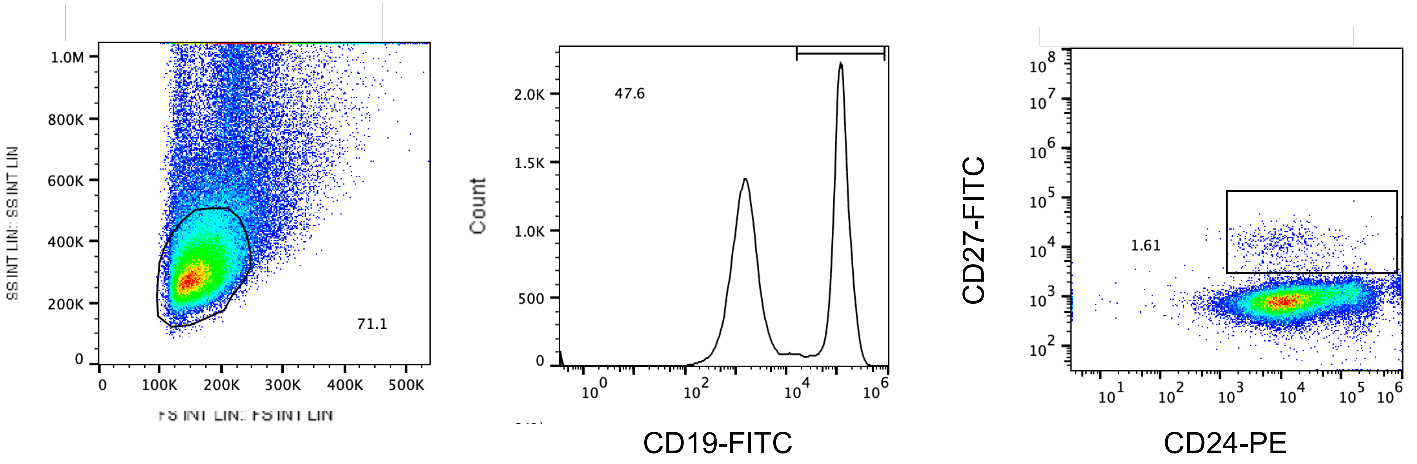

Bcell analysis CD40L with C21-4

Transitional Bcell

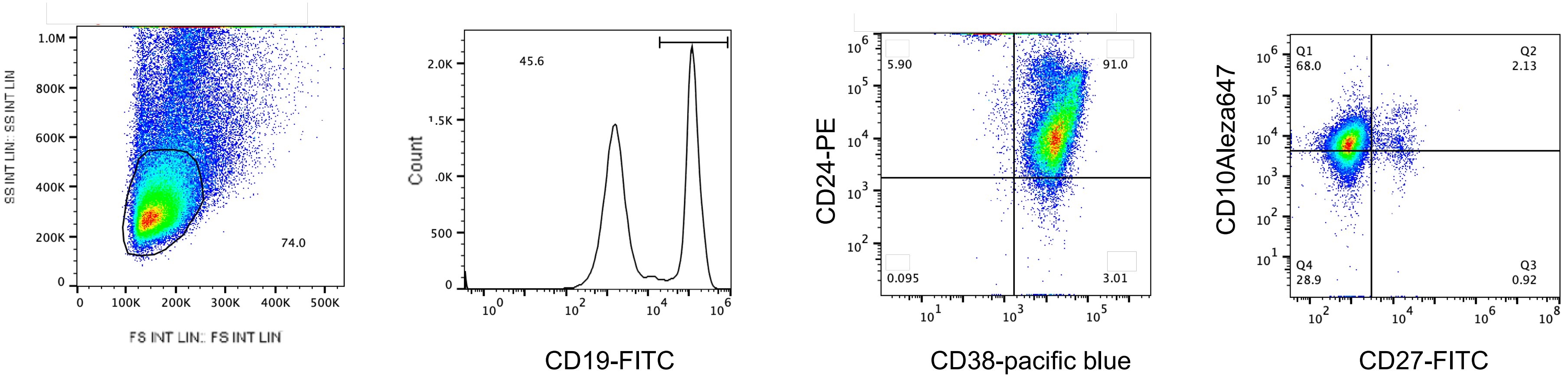

Plasmatic cell

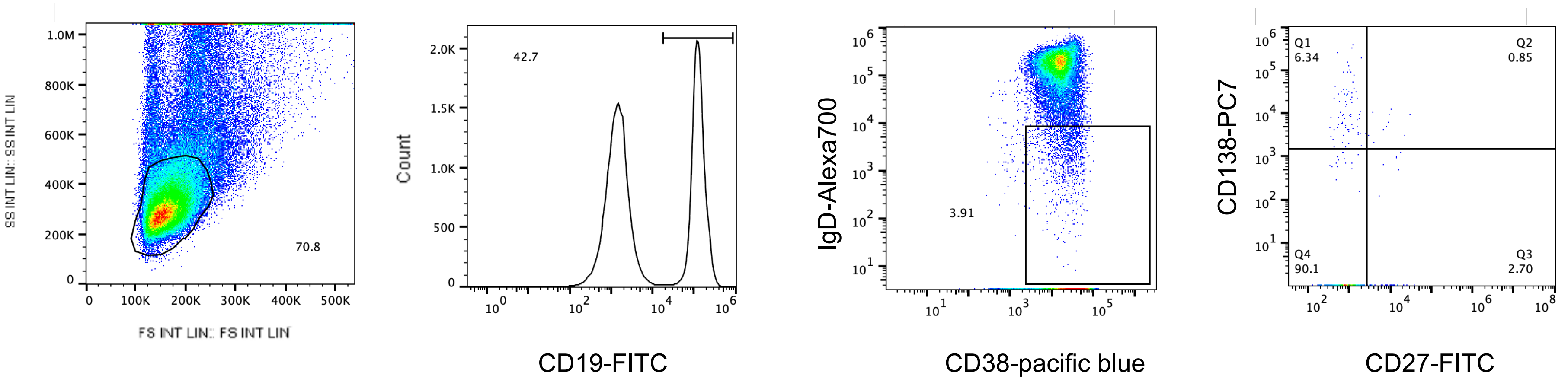

Bcell analysis CD40L with C21-5

Naïve Bcell

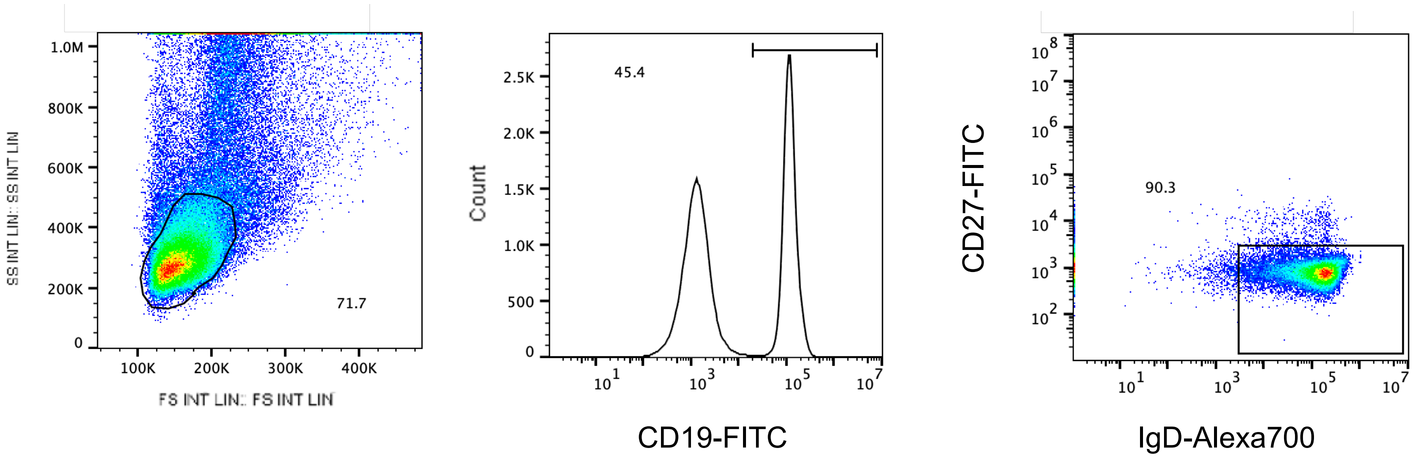

Memory Bcell

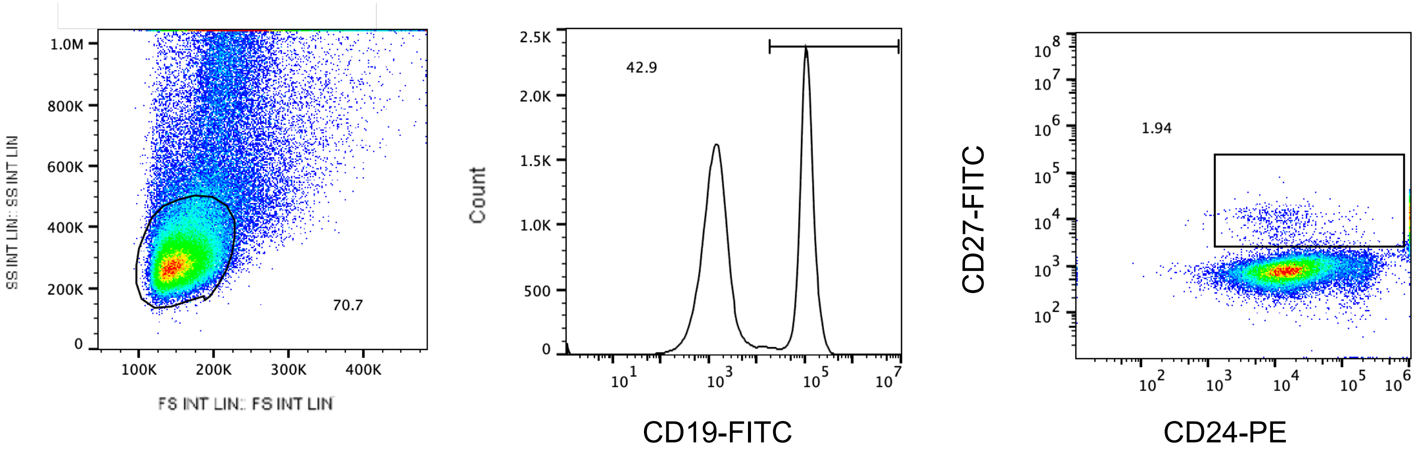

Bcell analysis CD40L with C21-5

Transitional Bcell

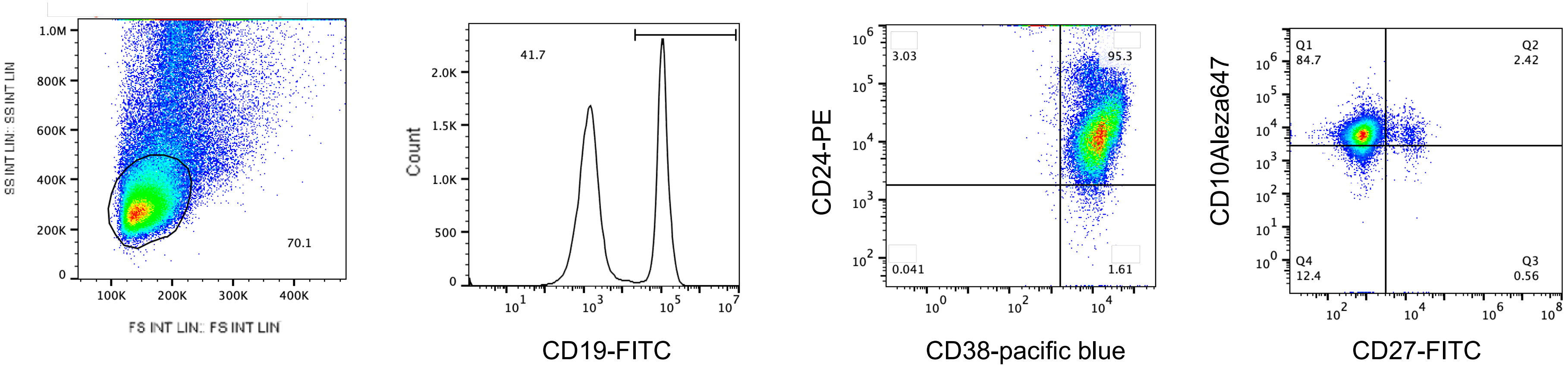

Plasmatic cell

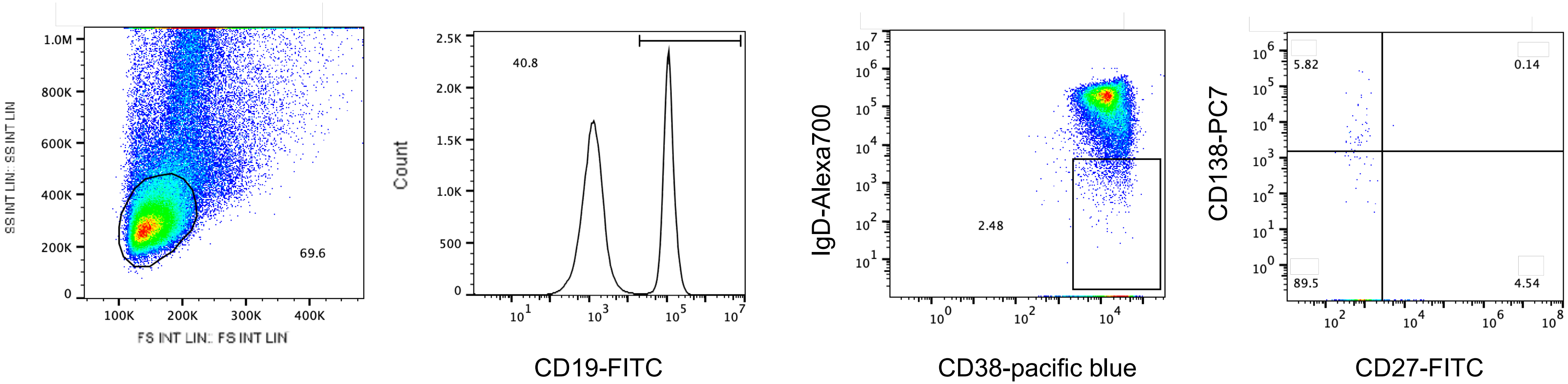

Bcell analysis LacZ-1

Naïve Bcell

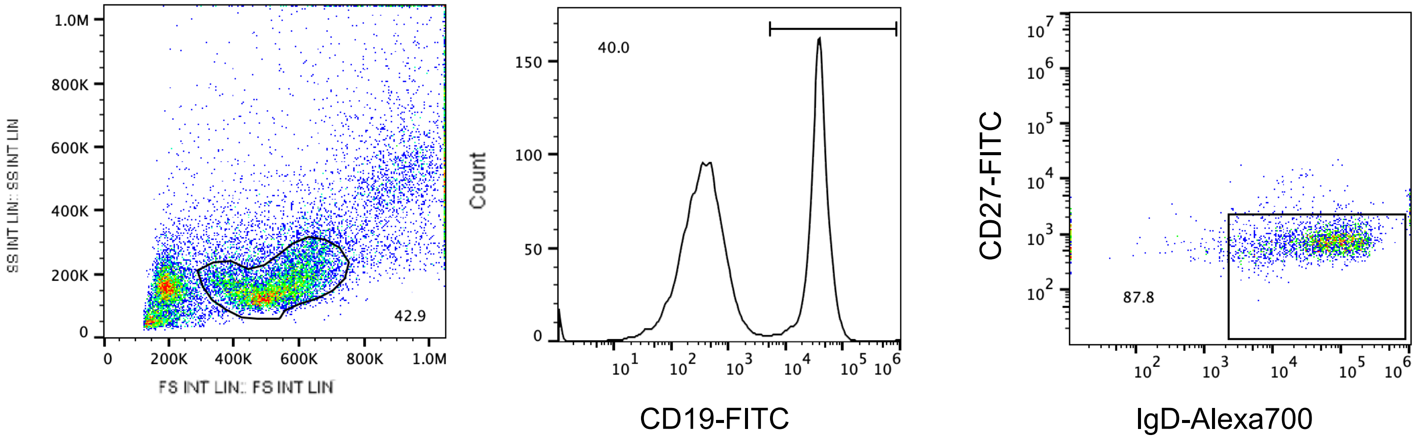

Memory Bcell

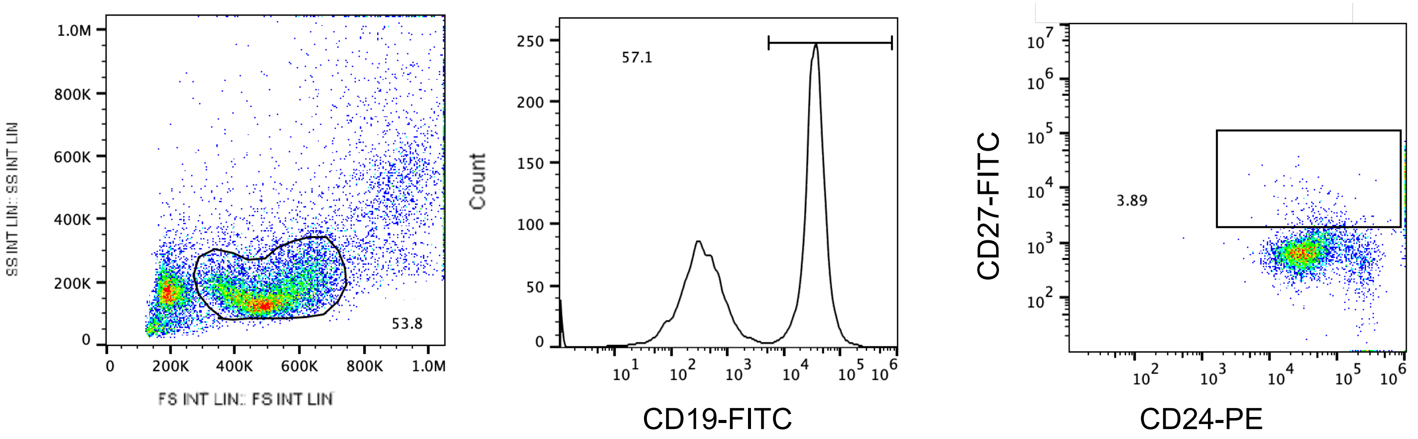

Bcell analysis LacZ-1

Transitional Bcell

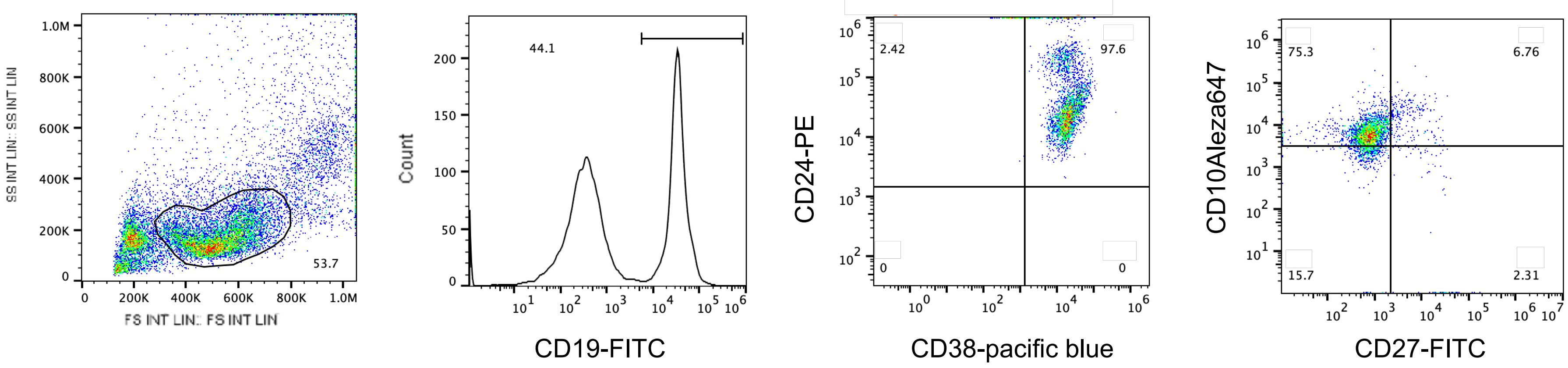

Plasmatic cell

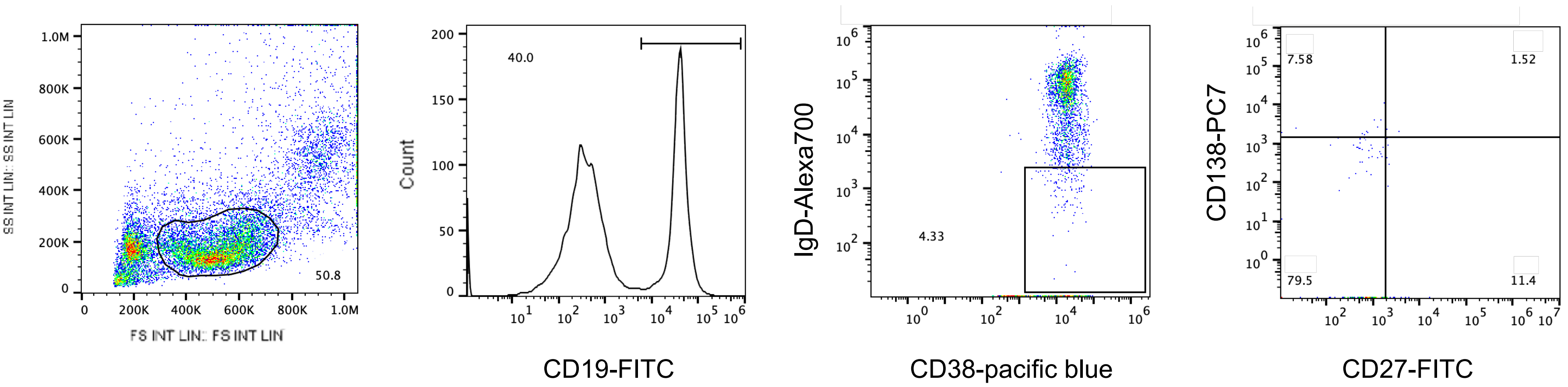

Bcell analysis LacZ-2

Naïve Bcell

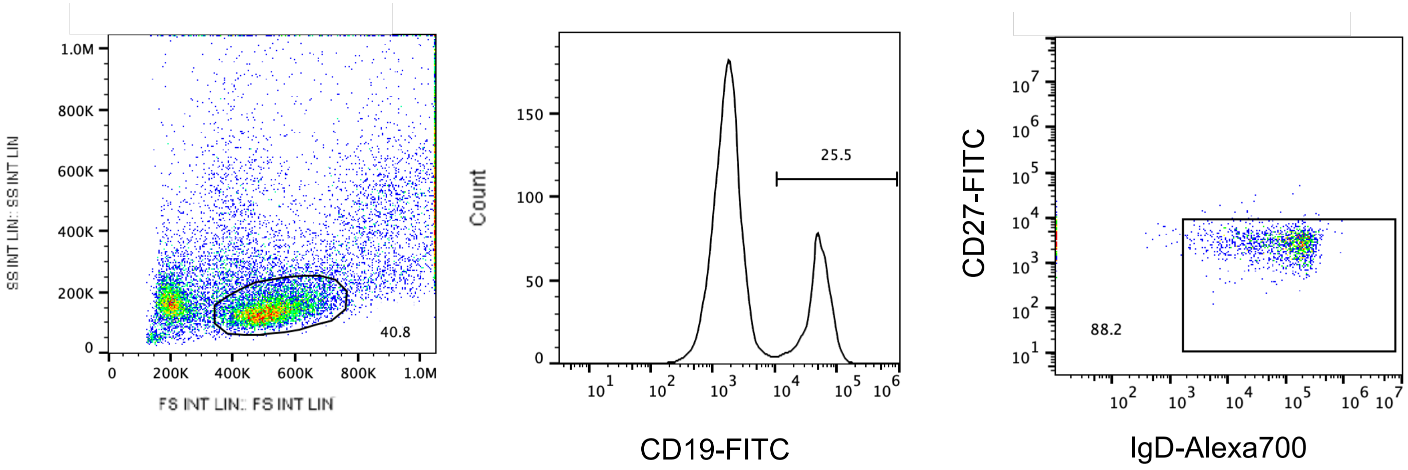

Memory Bcell

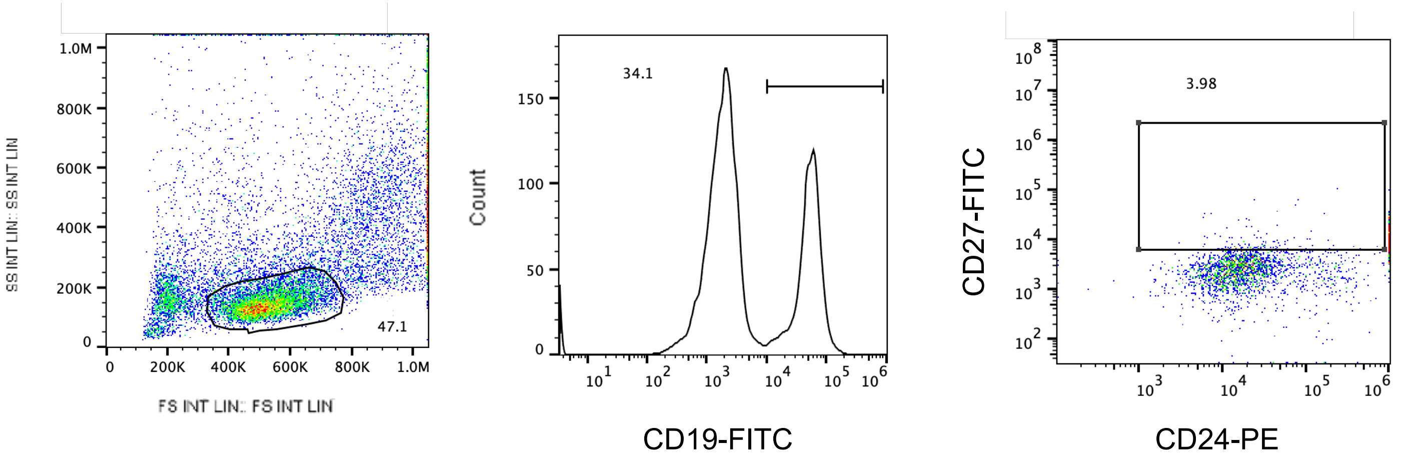

Bcell analysis LacZ-2

Transitional Bcell

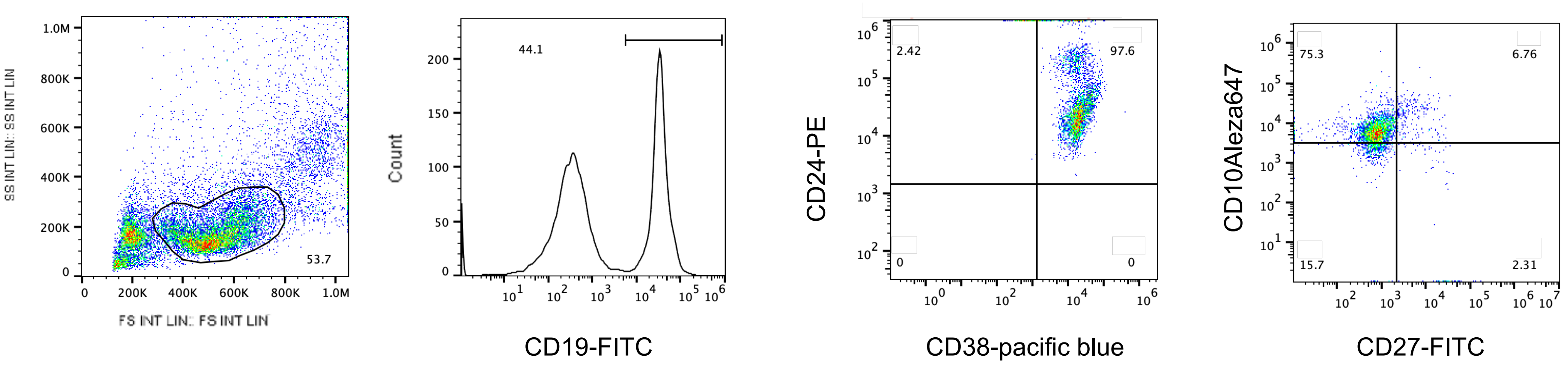

Plasmatic cell

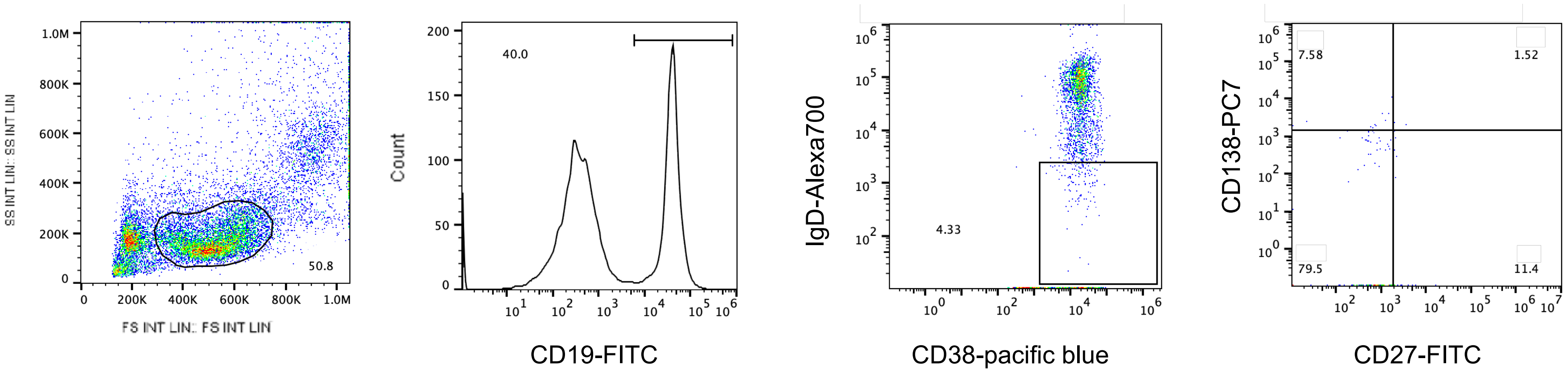

Bcell analysis LacZ-3

Naïve Bcell

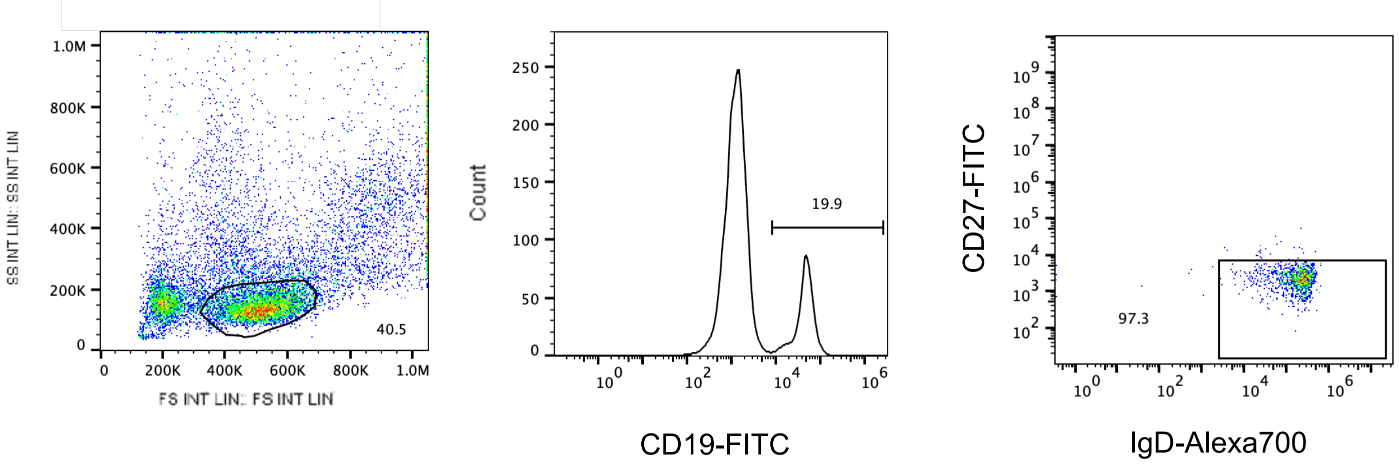

Memory Bcell

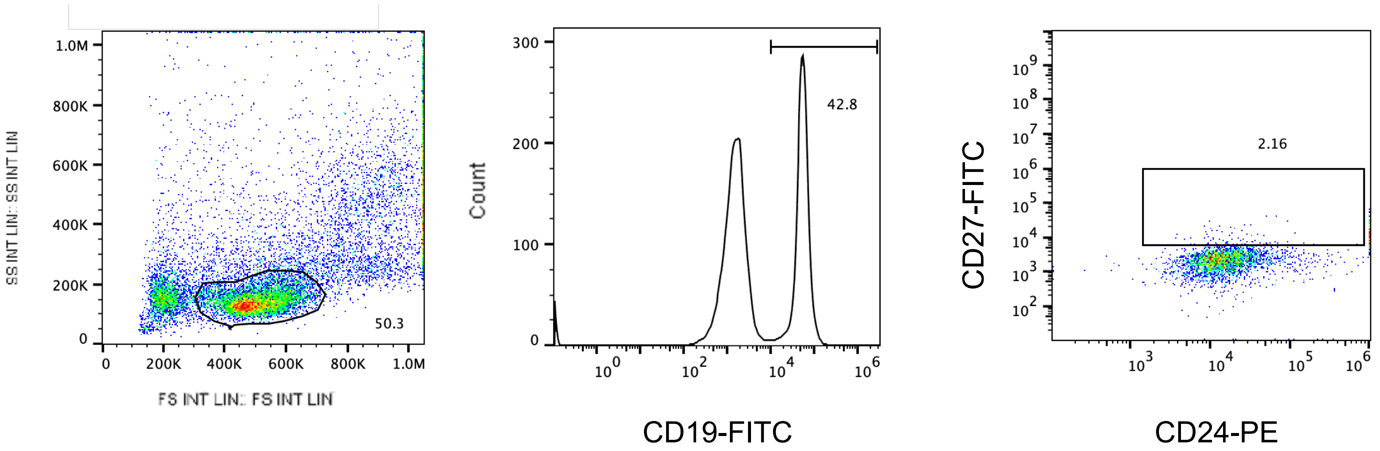

Bcell analysis LacZ-3

Transitional Bcell

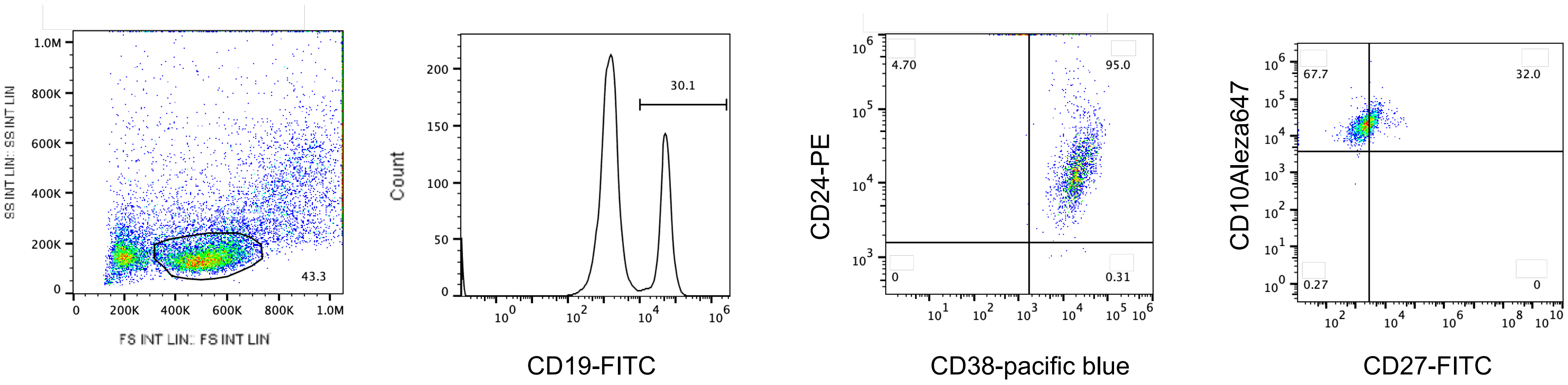

Plasmatic cell

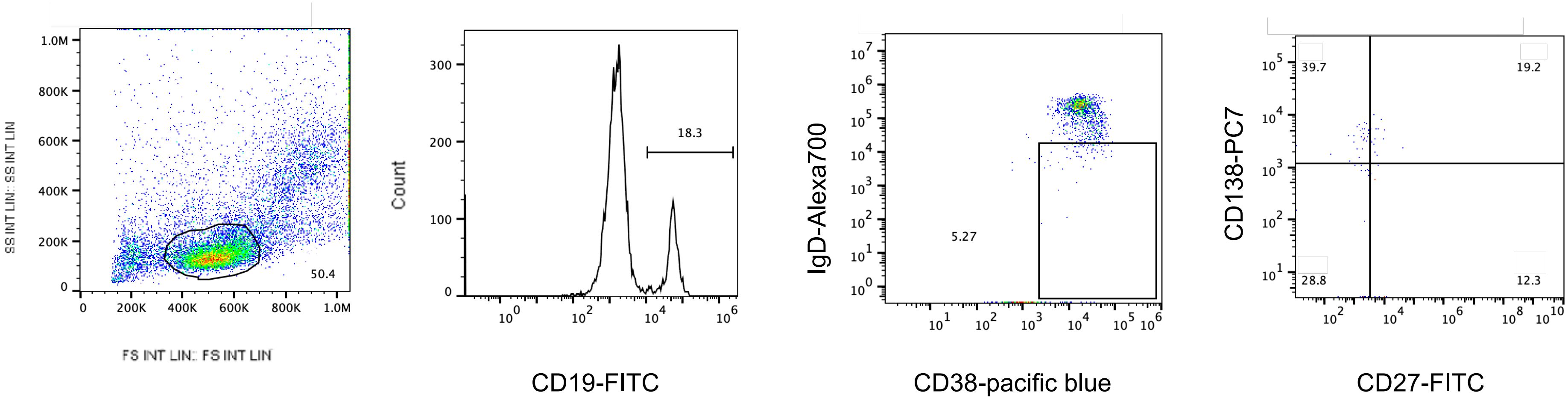

Bcell analysis LacZ-4

Naïve Bcell

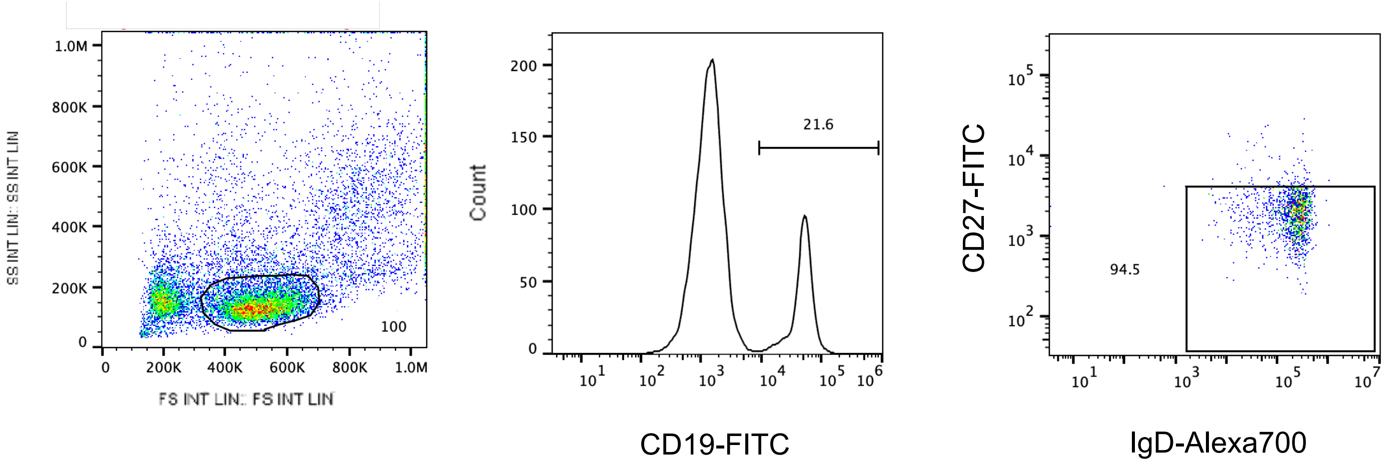

Memory Bcell

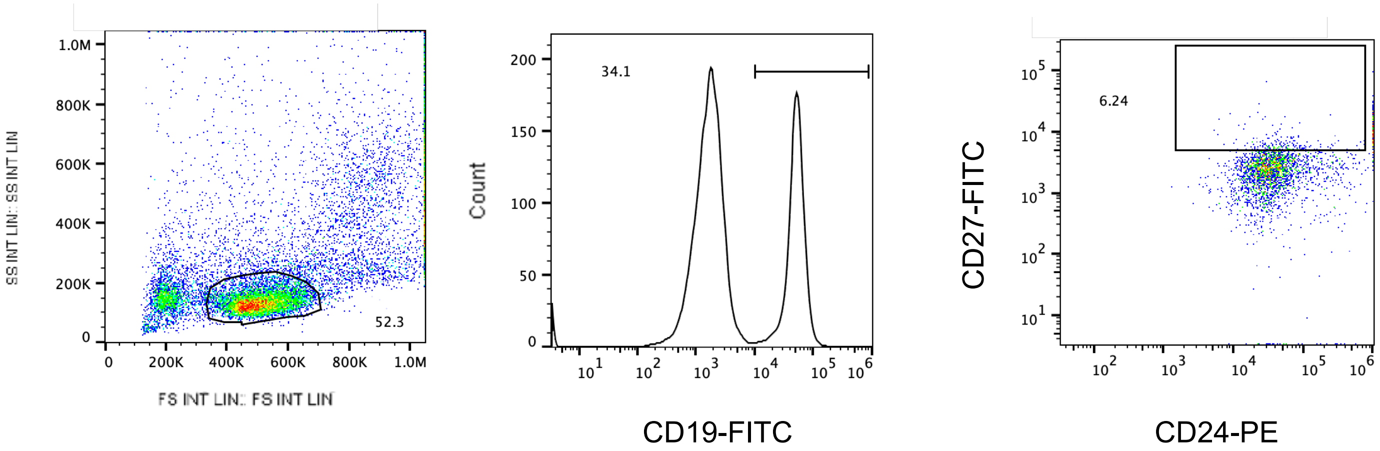

Bcell analysis LacZ-4

Transitional Bcell

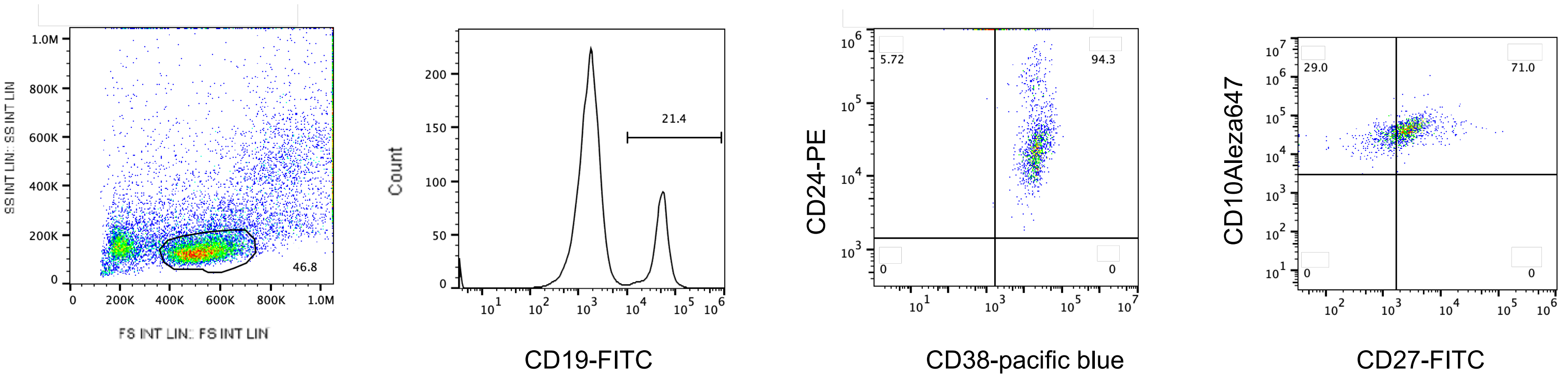

Plasmatic cell

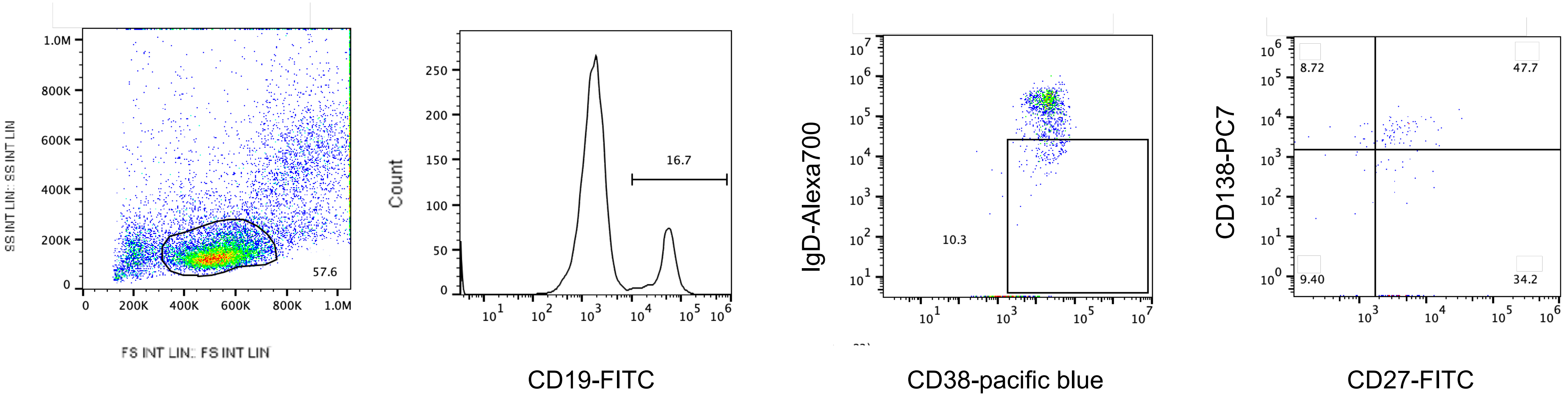

Supplement: Supplementary file 1 [file cimb-46-00579-s001.zip › Supplementary Figure S2.pdf]
